# Trends in Diet Quality by Race/Ethnicity among Adults in the United States for 2011–2018

**DOI:** 10.3390/nu14194178

**Published:** 2022-10-08

**Authors:** Meng-Hua Tao, Jia-Liang Liu, Uyen-Sa D. T. Nguyen

**Affiliations:** 1 Department of Public Health Sciences, Henry Ford Health System, Detroit, MI 48202, USA; 2 Department of Epidemiology and Biostatistics, College of Public Health, Temple University, Philadelphia, PA 19122, USA; 3 Department of Biostatistics and Epidemiology, University of North Texas Health Science Center, Fort Worth, TX 76107, USA

**Keywords:** diet quality, race/ethnicity, disparity, adults

## Abstract

This study aimed to investigate time trends in diet quality and the consumption of major food groups and nutrients by race/ethnicity among adults in the United States. Dietary data from 19,192 adults aged ≥ 20 years from four National Health and Nutrition Survey (NHANES) cycles (2011–2018) were included. The Healthy Eating Index (HEI) 2015 scores (range: 0–100; higher scores indicate better diet quality) and dietary consumption of food groups and nutrients were estimated for each cycle. Linear regression was used to test trends. For the overall population, the estimated overall HEI-2015 scores significantly decreased (*p* for trend = 0.011). However, decreases were observed in the estimated consumption of added sugars and total carbohydrates, while the estimated consumption of soy products and polyunsaturated fatty acids was significantly increased. A significant decrease in overall HEI-2015 score was observed in the non-Hispanic white group, but not in other racial/ethnic groups. Decreases in added sugar intake were found in the non-Hispanic black and Hispanic groups; sodium intake significantly decreased in the non-Hispanic Asian group. From 2011 to 2018, there was a decrease in estimated overall diet quality in US adults; however, there were improvements in certain nutrients and dietary components. Nevertheless, disparities in diet quality exist among racial/ethnic groups.

## 1. Introduction

Poor diet is a major contributor to chronic diseases, including obesity, type 2 diabetes, cardiovascular disease, liver disease, and some types of cancers [1,2,3,4]. In the United States (US), 60% of adults have one or more diet-related chronic diseases [5], and diet was one of the leading causes of death and one of the leading risk factors for disability-adjusted life years [6]. Therefore, it is crucial to understand trends in diet quality and corresponding disparities in the US population, which can provide important evidence to inform policy efforts and prevention programs aimed at improving diets and preventing diet-associated health consequences in the US population.

Many previous studies have generally investigated trends of dietary components (e.g., total energy, macronutrients, micronutrients) [7,8,9,10,11], focused on specific foods (e.g., processed meat, red meat, poultry, fish) [12,13], or used older diet data [14,15,16]. Limited evidence has been available on the contemporary trends in diet quality and the broad range of dietary factors, including trends related to multiple individual foods and nutrients among US adults across different racial/ethnic groups.

In the US, the Asian American population constituted the fastest growing racial/ethnic group between 2010 and 2020 [17], and the population is projected to surpass 46 million by 2060 [18]. Given the continuous growth of the US Asian population, there is an urgent need to better understand health conditions and risk factors in this population. Most recent data have shown significant increases in the prevalence of obesity and adiposity measures in non-Hispanic Asians between 2011 and 2018 [19]. However, current knowledge on trends and the national status relating to diet quality among Asian Americans compared to other racial/ethnic groups is not well established. To address these gaps, data from the National Health and Nutrition Examination Survey (NHANES) 2011–2018 were used to examine race/ethnicity-specific trends in overall diet quality and individual foods and nutrients among US adults.

## 2. Materials and Method

### 2.1. Study Population

Data from four continuous cycles of NHANES between 2011 and 2018 were utilized in this study. The NHANES is a cross-sectional survey designed to monitor health and nutrition in a nationally representative sample of the civilian, noninstitutionalized US population [20]. Detailed descriptions of the study have been reported elsewhere [20,21]. The NHANES data are released every two years by the U.S. National Center for Health Statistics (NCHS) of the Centers for Disease Control and Prevention (CDC) [20,21]. Beginning in 2011, NHANES oversampled NH Asians to increase precision for racial group estimates [20,21]. All participants provided written informed consent, and the Research Ethics Review Board at the NCHS approved the NHANES study protocol (Protocol #2011-17, 2018-01).

For this study, the study population included adults aged 20 years or older who had completed two valid 24-h dietary recalls during the four cycles of the NHANES from 2011–2012 through to 2017–2018 (*N* = 19,549). Women who were pregnant or lactating were further excluded from the analysis (*N*= 357). As a result, a total of 19,192 participants were included in the final analysis.

### 2.2. Assessment of Dietary Intake

Details of the protocol and dietary data collection methods have been fully described elsewhere [22]. Briefly, daily dietary intake information was obtained through 24-h recall interviews. From 2003, the NHANES collected two 24-h recalls for each participant using the US Department of Agriculture (USDA)’s Automated Multiple Pass Method (AMPM) of 5-step data collection. The first dietary recall was collected in person by trained interviewers in the NHANES mobile examination centers. The second dietary recall was completed by a telephone interview 3–10 days after the first recall.

To assess consumption of major food groups, the same definitions were used for the same food groups from the USDA Food Patterns Equivalents Database across different survey cycles [23,24,25]. Major nutrient intakes were calculated by using cycle-specific versions of the USDA food composition database, which has been described in detail elsewhere [26]. The USDA databases estimated the nutrient content of NHANES foods in recipes by linking the ingredients in the survey food recipes to food composition data [22]. Only dietary recall data verified as reliable by trained study staff were used in the analyses.

### 2.3. Assessments of Dietary Quality

Overall diet quality was assessed by the Healthy Eating Index (HEI) 2015, which measures adherence to the 2015–2020 Dietary Guidelines for Americans [27]. The foods and nutrients were represented on a density basis as amount per 1000 kcal. The HEI-2015 comprises nine adequacy components (total fruits, whole fruits, total vegetables, greens and beans, whole grains, dairy, total protein foods, seafood and plant proteins, and fatty acids) and four moderation components (refined grains, sodium, added sugars, and saturated fats) (Supplementary Method S1). The total HEI-2015 score ranged from 0 to 100, with higher scores indicating better diet quality (Supplementary Table S1). Secondary outcomes were trends related to the daily consumption of major food groups and nutrients consumed by US adults.

### 2.4. Other Variables

Information on age, sex, and race/ethnicity was collected during the in-person interviews. Information on race/ethnicity was self-reported via standardized survey questions on Hispanic origin and race. In NHANES 2011–2018, race/ethnicity was categorized as non-Hispanic (NH) white; NH black; Hispanic, referring to all Hispanics regardless of race; NH Asian, including all people with origins in any of the original peoples of the Far East, Southeast Asia, or the Indian subcontinent; and Other race, including American Indians or Alaska Natives, Native Hawaiians or other Pacific Islanders, and multiracial persons.

### 2.5. Statistical Analysis

All statistical analyses were conducted in SAS software (version 9.4, SAS Institute, Cary, NC, USA) using the “Survey” procedures to incorporate the complex multistage clustered probability sampling strategy of the NHANES. As the National Cancer Institute (NCI) and USDA recommended, we used the population ratio method to calculate the HEI-2015 score for each of the four cycles included in the study, both overall and by race/ethnicity. The population ratio method adjusts for day-to-day variation (also referred to as within-person variation) to derive a score that is closer to the usual intake at the population level [28]. Considering the day-to-day within-person variation in individual diets and the complex sample design of the NHANES, the NCI method was used in the current study to estimate usual dietary intake of nutrients and food groups in the overall population and by race/ethnicity [29]. Two 24-h dietary recalls were used while applying the NCI method (Supplementary Methods S2) [29]. Weighted means and 95% confidence intervals (CIs) were estimated for the HEI-215 score and intakes of major food groups and nutrients for each NHANES cycle. Intakes of all food groups and nutrients were energy-adjusted using the nutrient density method to evaluate trends in dietary quality, independent of the changes in energy intake during this period. We conducted trend analyses by treating the survey year as a continuous variable in the survey-weighted linear regression models. Absolute differences in estimated means with 95% CIs between the 2011–2012 and 2017–2018 cycles were calculated using the Welch–Satterthwaite procedure. All analyses were two-tailed, and significance was set at *p* < 0.05. No adjustments were made for multiple comparisons; the findings of secondary analyses should be interpreted as exploratory.

## 3. Results

Among the overall population in this study, the weighted mean age was 48.2 years; 9694 participants were women (weight proportion of 51.0%). From 2011 to 2018, the proportion of NH whites decreased from 67.1 to 63.0%, while the proportion of those in the Other category increased from 2.1 to 4.4%. The proportion of obesity and senior individuals (aged ≥ 65 years) increased from 35.1 to 43.9% and from 17.5 to 21.1%, respectively (Supplementary Table S2).

### 3.1. Healthy Eating Index 2015

For the overall population, the estimated mean of total HEI-2015 score decreased significantly from 55.01 (95% CI, 54.09–55.95) in 2011–2012 to 52.65 (95% CI, 51.12–54.19) (*p* for trend = 0.011 in 2017–2018) (Table 1). Statistically significant reductions in total HEI-2015 score were observed among NH whites (from 55.36 to 52.14; difference: −3.22, 95% CI, −5.40 to −1.04, *p* for trend = 0.007), as well as among NH black males (*p* for trend = 0.020). Among NH Asians, the estimated total HEI-2015 score remained stable, and was higher than any other racial/ethnic group across the survey cycles.

**Figure 1:**
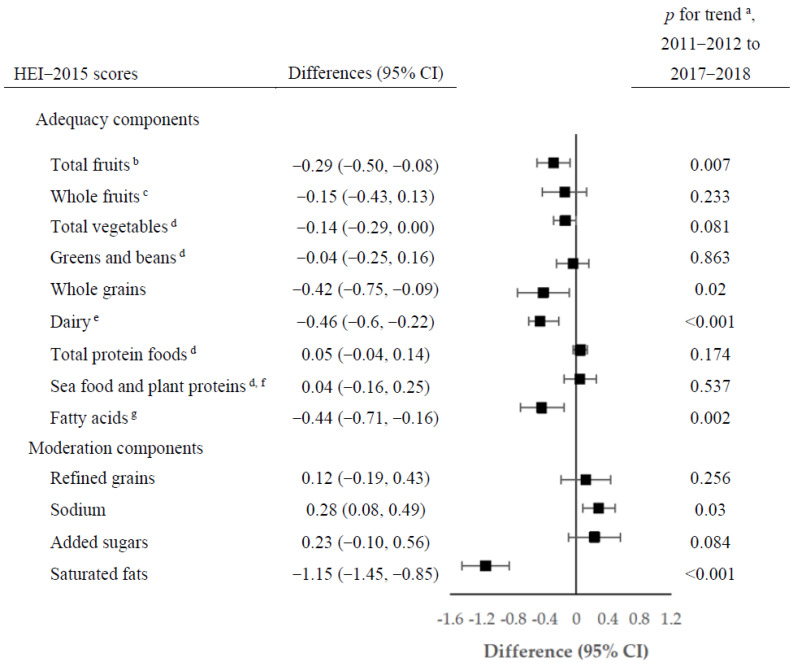
Changes in estimated scores of HEI-2015 components among US adults aged ≥ 20 years by NHANES cycles from 2011–2012 to 2017–2018. ^a^ *p* value from trend tests by modeling survey period as a continuous variable. ^b^ Includes 100% fruit juice. ^c^ Includes all forms except juice. ^d^ Includes legumes (beans and peas). ^e^ Includes all milk products, such as fluid milk, yogurt, and cheese, and fortified soy beverages. ^f^ Includes seafood, nuts, seeds, soy products (other than beverages), and legumes (beans and peas). ^g^ Ratio of PUFAs and MUFAs to SFAs ((PUFAs + MUFAs)/SFAs).

**Figure 2:**
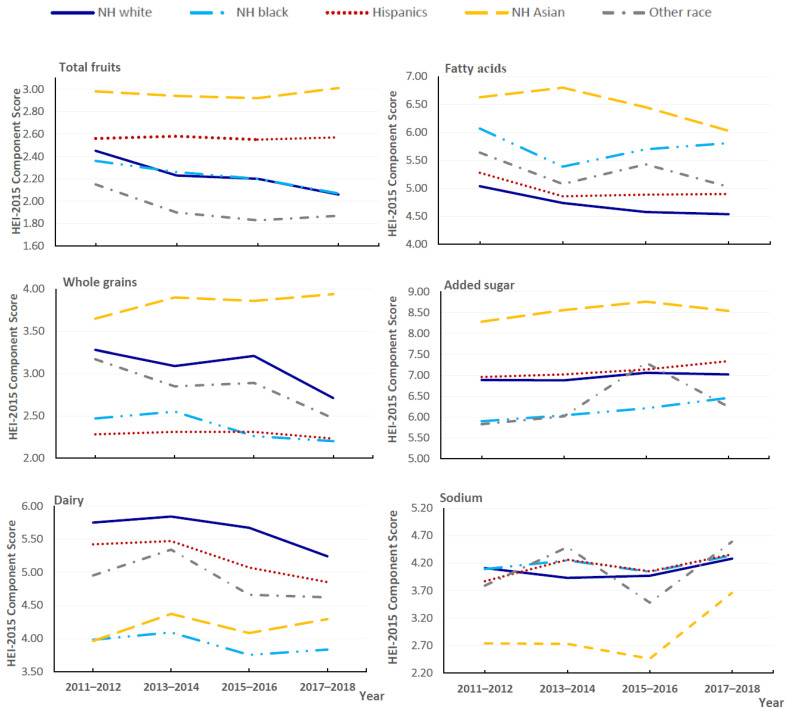
Trends in estimated scores of selected components of HEI-2015 by race/ethnicity among US adults aged ≥ 20 years for 2011–2018.

From 2011 to 2018, changes were also observed among individual component scores of the HEI-2015 in the overall population (Figure 1; Supplementary Table S3). The largest increase in the estimated component scores was observed for sodium (from 4.00 to 4.28; difference 0.28; 95% CI, 0.08–0.49; *p* for trend = 0.030), indicating reduced consumption of sodium. The largest reduction was observed for saturated fatty acids (from 6.41 to 5.26; difference −1.15; 95% CI, −1.45 to −0.85; *p* for trend < 0.001), corresponding to increased saturated fatty acids consumption. Statistically significant decreasing trends were observed for component scores for total fruits, whole grains, dairy, and fatty acids. Trends in selected components of the HEI-2015 scores by race/ethnicity are shown in Figure 2 (Supplementary Table S4). From 2011 to 2018, all racial/ethnic groups had improvements in the component score for sodium, particularly for the NH Asian group, which experienced the largest increase (from 2.74 to 3.66; difference 0.92; 95% CI, 0.56–1.28; *p* for trend <0.001). Moreover, the increases in the added sugar score were mainly observed in NH blacks (from 5.90 to 6.46; difference 0.56; 95% CI, 0.17–0.95; *p* for trend =0.005) and the Hispanic group. We observed statistically significant decreases in the component score for dairy in both Hispanic (*p* for trend = 0.003) and NH white groups (*p* for trend < 0.001); the estimated component scores for saturated fats decreased in all racial/ethnic groups. The component scores for total fruits and whole grains remained stable in most racial/ethnic groups, while statistically significant decreases were mainly present in the NH white group (*p* for trend = 0.007 and 0.012, respectively).

### 3.2. Trends in Specific Foods and Nutrients

Table 2 showed that, in the overall population from 2011 to 2018, the estimated mean daily consumption of added sugar significantly decreased from 16.35 to 15.53 tsp/2000 kcal (difference −0.81, 95% CI, −1.93 to 0.30; *p* for trend = 0.032). The estimated mean daily total fruits intake significantly decreased from 1.02 to 0.91 servings/2000 kcal (difference −0.10; 95% CI, −0.21 to 0.001; *p* for trend = 0.005), which may be due to reduced consumption of 100% fruit juices (*p* for trend < 0.001). Similarly, statistically significant decreases were found for the mean daily intake of total grains, whole grains, total dairy, and milk products. Meanwhile, there were statistically significant increases in the estimated mean daily consumption of soy products and eggs. Among nutrients, the estimated mean daily consumption significantly decreased for carbohydrates (difference, −13.09; 95% CI, −16.97 to −9.20; *p* for trend < 0.001) and fiber (difference −1.05; 95% CI, −1.88 to −0.22; *p* for trend = 0.016). Moreover, the estimated mean intakes were significantly increased for saturated, monounsaturated, and polyunsaturated fatty acids, as well as cholesterol (*p* for trend < 0.001 for all). The estimated daily intakes of total vegetables, refined grains, legumes, processed meat, unprocessed red meat, poultry, fish/sea food, and nuts and seeds did not significantly change.

**Figure 3:**
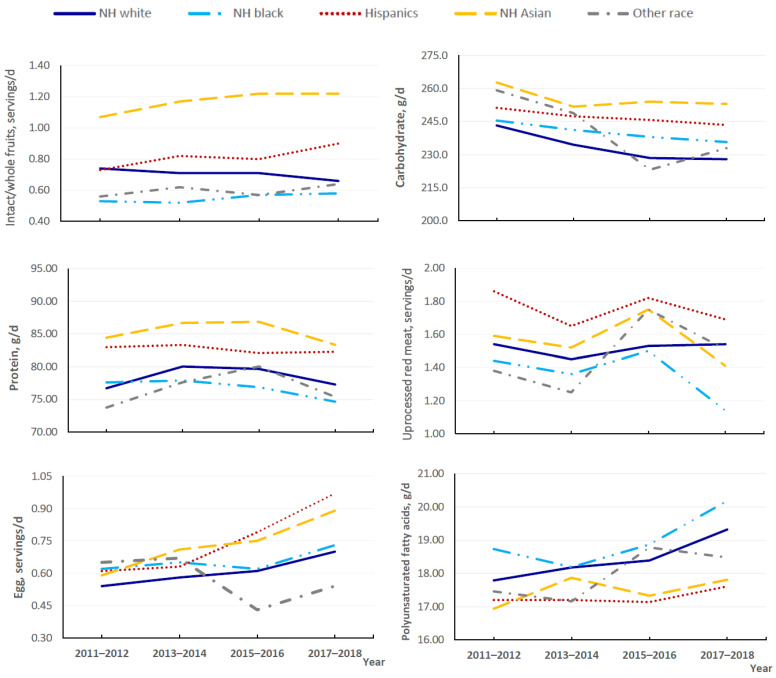
Trends in estimated mean daily consumption of selected food groups and nutrients by race/ethnicity among US adults aged ≥ 20 years for 2011–2018.

Trends in the consumption of individual food groups and nutrients also showed notable variations across racial/ethnic groups (Figure 3; Supplementary Table S5). For example, consumption of total fruits and intact/whole fruit significantly decreased among NH white individuals, while remaining stable in other racial/ethnic groups. Moreover, statistically significant decreases in the consumption of total meat, unprocessed red meat, and processed meat were limited in the NH black group (*p* for trend < 0.05 for all). Increases in egg intake were mainly observed in the Hispanic, NH Asian, and NH white groups, but total dairy intake significantly decreased in the NH white and Hispanic groups. We observed significant reductions in added sugar intake in the NH black and Hispanic groups (*p* for trend < 0.05 for all). The estimated mean sodium intake significantly decreased in NH Asian adults (difference, −256.45 mg/d; 95% CI, −384.65 to −128.21; *p* for trend = 0.009), although it is still higher than intakes in other racial/ethnic groups.

## 4. Discussion

Based on the nationally representative data collected in a multi-racial/ethnic population between 2011–2012 and 2017–2018, this study estimated the most recent national trends regarding diet quality, as well as their racial/ethnic disparities among US adults. Overall diet quality as assessed by the HEI-2015 decreased, with declines in the consumption of fruits (especially fruit juice), whole grains (primarily whole grains), and total dairy (primarily milk products) and an increased intake of saturated fatty acids. However, several improvements in the US diet were identified. Consistent with concurrent Dietary Guidelines for Americans [5], US adults decreased their intake of added sugars and carbohydrates and increased their consumption of soy products and polyunsaturated fatty acids. Moreover, there were variations in overall diet quality and the consumption of specific food groups and nutrients across racial/ethnic groups. No significant trends were identified for the consumption of total vegetables, poultry, legumes, or nuts and seeds, while small changes were observed for other food components across racial/ethnic groups.

Consistent with previous reports in US adults [8,15], our results showed similar disparities in diet quality by race/ethnicity, and trends for overall diet quality differed significantly across racial/ethnic groups. For example, the NH Asian group was plateauing at a high score in relation to overall HEI-2015 score between 2011 and 2018 but had the most notable increases for sodium component score. The NH black group had worse overall diet quality, as measured by the HEI-2015, than other racial/ethnic groups while showing an improvement in the component score for added sugar. Similarly, Hispanics also showed an increase in the component score for added sugar. NH whites were the only group with a statistically significant decrease in overall HEI-2015 score.

Sweetened beverages, mainly soft drinks, are the number one source of added sugars among US adults; fruit drinks rank as the second largest source of added sugars among NH blacks and Hispanics [30]. Consistent with results among US youth [31], our results showed decreases in 100% fruit juice intake for most racial/ethnic groups, which may be one contributor to the observed declines in intakes of added sugars among US adults, particularly those in the Hispanic and NH black groups. Consistent with previous reports among US adults [32], we observed significant increases in egg consumption in most racial/ethnic groups, except among NH blacks, which may be owed to the removal of dietary egg restrictions from the Dietary Guidelines for Americans [33].

Previous studies reported that Asians, especially people in East and Central Asia, have the highest sodium consumption in the world [34,35]. However, sodium intake status among Asian Americans was not well understood [36]. From 2011 to 2018, we observed a significant decline in estimated mean sodium intake in the NH Asian group, which may be related to the increased awareness of the harmful health effects of excess sodium [37]. Yet, salt, soy sauce, bean paste, fish, or shrimp sauce, the top sources of sodium in traditional Asian diets, are very often added during cooking [36,38]. To achieve the Dietary Guidelines for Americans’ recommended sodium limit [5], it is necessary to better understand sodium intake (e.g., amount and pattern) and its long-term trend among Asian Americans so that more targeted intervention and education programs on cooking or eating behaviors can be developed for this minority group.

Despite the aforementioned improvements, important dietary challenges remain. First, US adults did not meet the 2020 Dietary Guidelines for Americans’ recommendation for dairy intake [5], with significant decreases in the consumption of dairy products, such as milk products, among people in the NH white and Hispanic groups. Second, Americans are advised to limit the amount of saturated fat in their diets [5]. In contrast to decreases in total meat intake in the NH black, Hispanic, and NH Asian groups, and reductions in the consumption of unprocessed red meat and processed meat among NH blacks, statistically significant increases in saturated fat intake were observed in all racial/ethnic groups from 2011 to 2018. The underlying reasons are unclear and warrant further exploration. Third, although the mean daily carbohydrate intake significantly decreased among NH Asian adults, they still had the highest intake of carbohydrates. Previous studies reported that refined carbohydrates, including white rice and white flour, remain as mainstays in Asian American diets [38,39], whereas another study showed that greater acculturation was associated with a decreased intake of white rice in East Asian immigrants [40]. Nevertheless, more targeted health education is needed in Asian American communities to improve knowledge of the health risks of a high carbohydrate and refined grains diet and to promote changes in diet behaviors.

The main strength of this study is the use of the most recent dietary data from the NHANES with a sufficiently large sample size of minority groups, including NH Asian subjects, which allows us to show the distinct but neglected trends of diet quality among Asian Americans. This study has several limitations. First, the NH Asian group is a heterogeneous population with diverse ethnic origins, socioeconomic statuses, dietary behaviors and other lifestyle factors, cultural beliefs and behaviors, immigration experience, and acculturation. However, data on NH Asians was aggregated as one racial/ethnic group in the NHANES without consideration of ethnic origins; thus, we were unable to test the trends for ethnic groups included as NH Asians. Second, self-reported 24-h dietary recalls may result in both random and systematic errors while potentially suffering from recall bias [41]. However, the NCI method that uses data from two 24-h recalls was applied to reduce measurement errors and improve estimates of the usual intake among a population [42]. Third, we conducted trend tests using data from the most recent four cycles, which prevented us from making conclusions regarding the longer-term trends.

In conclusion, results from this study showed variations in trends related to diet quality across race/ethnicity, with a decrease in overall diet quality in the NH white group from 2011 to 2018. We observed significant decreases in the consumption of added sugar among NH blacks and Hispanics. Additionally, people in the NH Asian group continued to have a high-quality diet, with a decrease in sodium intake. Findings from the analyses of nationally representative surveys provide an important picture of recent trends in diet quality, including individual food groups and nutrients in US adults, and may inform future opportunities to develop targeted prevention approaches aimed at improving diet quality for different racial/ethnic groups in the United States.

## Figures and Tables

**Table 1:** Trends in estimated HEI-2015 score among adults aged ≥ 20 years by race/ethnicity and sex, NHANES, 2011–2018 *.

Total HEI-2015 Scores	Survey-Weighted Mean Score (95% CI)					
	2011–2012 (*n* = 4313)	2013–2014 (*n* = 4559)	2015–2016 (*n* = 4394)	2017–2018 (*n* = 4058)	Differences 2017–2018 vs. 2011–2012 (95% CI)	*p* for Trend ^a^
Overall	55.01 (54.09, 55.95)	54.18 (53.46, 54.91)	53.91 (52.46, 55.36)	52.65 (51.12, 54.19)	−2.36 (−4.16, −0.57)	0.011
All participants						
NH white	55.36 (54.19, 56.53)	54.03 (53.21, 54.86)	54.15 (52.50, 55.80)	52.14 (50.30, 53.98)	−3.22 (−5.40, −1.04)	0.007
NH black	52.48 (50.27, 54.70)	51.75 (50.57, 50.92)	50.63 (48.67, 52.59)	50.82 (49.04, 52.59)	−1.67 (−4.50, 1.17)	0.176
Hispanic	54.06 (52.91, 55.21)	54.58 (52.64, 56.52)	52.75 (51.28, 54.21)	54.04 (52.10, 55.99)	−0.02 (−2.28, 2.25)	0.625
NH Asian	59.85 (58,09, 61.61)	61.16 (59.54, 62.79)	60.52 (58.89, 62.16)	59.85 (57.36, 62.33)	−0.01 (−3.05, 3.04)	0.855
Other race ^b^	52.83 (48.68, 56.38)	52.36 (48.10, 56.63)	53.57 (50.12, 57.03)	50.60 (46.84, 54.35)	−1.93 (−7.31, 3.44)	0.498
Male						
NH white	54.08 (53.14, 55.02)	52.01 (51.01 53.02)	53.40 (51.07, 55.73)	50.46 (48.65, 52.27)	−3.62 (−5.66, −1.58)	0.007
NH black	51.88 (49.27, 54.49)	51.41 (50.40, 52.43)	48.80 (46.99, 50.61)	48.77 (46.89, 50.65)	−3.11 (−6.32, 0.11)	0.020
Hispanic	52.64 (51.22, 54.07)	53.17 (50.15, 56.20)	50.61 (49.33, 51.89)	52.30 (50.39, 54.22)	−0.34 (−2.73, 2.05)	0.379
NH Asian	59.31 (57.01, 61.60)	60.88 (58.06, 63.69)	59.14 (56.91, 61.36)	58.98 (55.76, 62.20)	−0.33 (−4.28, 3.63)	0.643
Other race ^b^	48.71 (44.42, 53.00)	48.31 (44.15, 52.48)	50.96 (47.57, 54.35)	53.33 (47.55, 59.11)	4.62 (−2.58, 11.82)	0.137
Female						
NH white	56.61 (54.88, 58.34)	56.01 (55.11, 56.91)	54.90 (52.91, 56.89)	53.72 (51.61, 55.84)	−2.89 (−5.62, −0.15)	0.025
NH black	52.95 (50.87, 55.02)	52.01 (50.24, 53.78)	52.23 (49.93, 54.53)	51.73 (50.49, 54.97)	−0.22 (−3.27, 2.84)	0.928
Hispanic	55.55 (54.24, 56.86)	56.00 (54.41, 57.59)	54.78 (52.46, 57.11)	55.70 (53.49, 57.92)	0.16 (−2.42, 2.73)	0.869
NH Asian	60.38 (57.90, 62.86)	61.45 (59.41, 63.49)	61.89 (59.73, 64.05)	60.64 (58.02, 63.26)	0.26 (−3.35, 3.87)	0.874
Other race ^b^	56.28 (51.60, 60.95)	57.14 (52.04, 62.23)	55.89 (51.06, 60.71)	47.55 (45.69, 49.41)	−8.73 (−13.76, −3.69)	<0.001

* Abbreviation: NHANES, National Health and Nutrition Examination Survey; NH, Non-Hispanic. ^a^ *p* value obtained from trend tests by modeling the survey period as a continuous variable. ^b^ Other race: includes race/ethnicity other than NH white, NH black, Hispanic, or NH Asian, including multiracial persons.

**Table 2:** Trends in estimated mean consumption of food groups and nutrients among adults aged ≥ 20 years, NHANES 2011–2018.

Foods/Nutrients	Survey-Weighted Mean Score (95% CI)					
	2011–2012 (*n* = 4313)	2013–2014 (*n* = 4559)	2015–2016 (*n* = 4394)	2017–2018 (*n* = 4058)	Differences 2017–2018 vs. 2011–2012 (95% CI)	*p* for Trend ^a^
Food density (per 2000 kcal per day)						
Total fruits (servings)	1.02 (0.93, 1.10)	0.96 (0.89, 1.04)	0.97 (0.89, 1.04)	0.91 (0.83, 0.99)	−0.10 (−0.21, 0.001)	0.005
Intact/whole fruit	0.73 (0.66, 0.79)	0.72 (0.66, 0.79)	0.74 (0.65, 0.82)	0.72 (0.64, 0.79)	−0.01 (−0.10, 0.09)	0.230
100% fruit juices	0.30 (0.26, 0.34)	0.26 (0.24, 0.28)	0.26 (0.23, 0.28)	0.21 (0.19, 0.23)	−0.09 (−0.14, −0.05)	<0.001
Total vegetables (servings)	1.65 (1.55, 1.75)	1.56 (1.49, 1.64)	1.64 (1.54, 1.73)	1.60 (1.51, 1.70)	−0.05 (−0.18, 0.08)	0.463
Total grains (servings)	6.28 (6.14, 6.43)	6.21 (6.09, 6.34)	6.07 (5.99, 6.15)	6.13 (5.95, 6.31)	−0.15 (−0.36, 0.06)	0.025
Whole grains	0.99 (0.88, 1.09)	0.93 (0.87, 0.98)	0.94 (0.87, 1.00)	0.80 (0.70, 0.90)	−0.19 (−0.32, −0.05)	0.008
Refined grains	5.31 (5.18, 5.45)	5.30 (5.16, 5.43)	5.14 (5.05, 5.23)	5.32 (5.19, 5.45)	0.01 (−0.16, 0.18)	0.535
Legumes (servings)	0.12 (0.10, 0.14)	0.11 (0.10, 0.12)	0.12 (0.10, 0.13)	0.11 (0.09, 0.13)	−0.01 (−0.04, 0.01)	0.740
Soy products (servings)	0.07 (0.05, 0.09)	0.08 (0.06, 0.10)	0.12 (0.10, 0.15)	0.11 (0.08, 0.15)	0.04 (0.01, 0.08)	0.004
Total meat (servings)	4.59 (4.41, 4.77)	4.79 (4.54, 5.03)	4.76 (4.53, 4.98)	4.61 (4.42, 4.80)	0.02 (−0.22, 0.26)	0.726
Unprocessed red meat	1.57 (1.39, 1.75)	1.47 (1.38, 1.56)	1.59 (1.49, 1.68)	1.50 (1.32, 1.69)	−0.07 (−0.30, 0.17)	0.525
Processed meat	0.94 (0.87, 1.01)	0.98 (0.88, 1.07)	0.96 (0.89, 1.04)	0.93 (0.85, 1.02)	−0.01 (−0.11, 0.10)	0.276
Poultry	1.44 (1.29, 1.59)	1.65 (1.51, 1.79)	1.56 (1.39, 1.72)	1.51 (1.37, 1.65)	0.08 (−0.11, 0.27)	0.116
Fish/seafood	0.63 (0.49, 0.87)	0.69 (0.55, 0.84)	0.61 (0.52, 0.69)	0.63 (0.52, 0.73)	−0.002 (−0.16, 0.16)	0.369
Fish high in omega-3 fatty acids	0.16 (0.11, 0.20)	0.22 (0.17, 0.26)	0.19 (0.15, 0.24)	0.16 (0.12, 0.20)	0.003 (−0.05, 0.06)	0.510
Fish low in omega-3 fatty acids	0.46 (0.36, 0.56)	0.48 (0.35, 0.62)	0.42 (0.34, 0.51)	0.48 (0.38, 0.57)	0.02 (−0.11, 0.14)	0.439
Eggs (servings)	0.56 (0.51, 0.61)	0.60 (0.56, 0.65)	0.64 (0.60, 0.69)	0.75 (0.66, 0.83)	0.18 (0.09, 0.28)	<0.001
Total dairy (servings)	1.50 (1.44, 1.55)	1.50 (1.44, 1.57)	1.41 (1.37, 1.46)	1.35 (1.29, 1.40)	−0.15 (−0.22, −0.08)	<0.001
Milk products (servings)	0.73 (0.68, 0.78)	0.68 (0.64, 0.71)	0.62 (0.58, 0.65)	0.59 (0.53, 0.64)	−0.14 (−0.21, −0.07)	<0.001
Cheese (servings)	0.68 (0.63, 0.72)	0.72 (0.67, 0.77)	0.67 (0.64, 0.71)	0.68 (0.64, 0.73)	0.01 (−0.05, 0.06)	0.336
Nuts and seeds (servings)	0.72 (0.63, 0.80)	0.72 (0.63, 0.81)	0.73 (0.61, 0.86)	0.76 (0.63, 0.89)	0.04 (−0.10, 0.18)	0.221
Added sugars (tsp)	16.35 (15.57, 17.12)	15.95 (15.31, 16.59)	15.11 (14.51, 15.70)	15.53 (14.58, 16.48)	−0.81 (−1.93, 0.30)	0.032
Nutrients (per day)						
Carbohydrate (g)	246.02 (242.99, 249.05)	238.47 (235.84, 241.10)	233.38 (229.98, 236.79)	232.94 (229.97, 235.91)	−13.09 (−16.97, −9.20)	<0.001
Protein (g)	78.03 (76.86, 79.20)	80.55 (78.45, 82.65)	80.14 (78.68, 81.60)	78.02 (76.46, 79.58)	−0.01 (−1.79, 1.77)	0.570
Saturated fat (g)	23.51 (22.91, 24.11)	24.39 (24.04, 24.74)	25.70 (25.20, 26.21)	26.13 (25.57, 26.68)	2.62 (1.87, 3.37)	<0.001
Monounsaturated fat (g)	26.26 (25.82, 26.71)	26.41 (26.12, 26.70)	27.80 (27.29, 28.31)	27.46 (26.90, 28.03)	1.20 (0.54, 1.86)	<0.001
Polyunsaturated fat (g)	17.76 (17.47, 18.06)	17.99 (17.64, 18.35)	18.21 (17.77, 18.65)	19.02 (18.36, 19.69)	1.26 (0.59, 1.92)	<0.001
Omega-3 fatty acids (g)	0.09 (0.08, 0.10)	0.10 (0.08, 0.11)	0.10 (0.09, 0.10)	0.09 (0.08, 0.10)	0.0002 (−0.01, 0.01)	0.771
Cholesterol (g)	265.04 (258.25, 271.84)	280.50 (273.96, 287.05)	292.47 (282.91, 302.02)	295.34 (281.75, 308.93)	30.30 (16.38, 44.22)	<0.001
Sodium (mg)	3374.22 (3352.96, 3395.49)	3393.99 (3346.66, 3441.32)	3433.22 (3373.58, 3492.86)	3372.48 (3286.77, 3459.19)	−1.74 (−83.73, 80.05)	0.609
Fiber (g)	17.36 (16.69, 18.03)	16.77 (16.20, 17.34)	16.99 (16.34, 17.64)	16.31 (15.70, 16.91)	−1.05 (−1.88, −0.22)	0.016

^a^ *p* value from obtained from trend tests by modeling the survey period as a continuous variable.

## Data Availability

Publicly available datasets were analyzed in this study. This data can be found here: https://www.cdc.gov/nchs/nhanes/ (accessed on 19 September 2022).

## Supplementary Materials

The following supporting information can be downloaded at: https://www.mdpi.com/article/10.3390/nu14194178/s1, Methods S1: The Health Eating Index (HEI) 2015, Methods S2: NCI Method, Table S1: Dietary components of Health Eating Index (HEI) 2015 and scoring standards, Table S2: Demographics of adults aged ≥ 20 years by National Health and Nutrition Examination Survey (NHANES) cycles, 2011–2018, Table S3: Trends in estimated HEI-2015 among adults aged ≥ 20 years by NHANES cycles from 2011 to 2018, Table S4: Trends in estimated HEI-2015 by race/ethnicity among adults aged ≥ 20 years, NHANES 2011–2018, Table S5: Trends in estimated mean consumption of selected food groups and nutrients by race/ethnicity among adults aged ≥ 20 years, NHANES 2011–2018 [28,42,43,44,45,46].
