# Supplementary material for: Trends in Diet Quality by Race/Ethnicity among Adults in the United States for 2011–2018
Source: Nutrients. 2022 Oct 8;14(19):4178. doi: 10.3390/nu14194178 (PMC9570938; doi:10.3390/nu14194178)
Supplement: Supplementary file 1 [file nutrients-14-04178-s001.zip › nutrients-1954320-supplementary.pdf]

**Supplementary Material**

## Methods S1.

### *The Health Eating Index (HEI) 2015*

The HEI is a measure of diet quality, independent of quantity, that can be used to assess compliance with the U.S. Dietary Guidelines for Americans (DGA). The original HEI was developed in 1995 by the US Department of Agriculture's (USDA) Center for Nutrition Policy and promotion, and since 2005 it has been periodically updated through a collaboration between USDA and the National Cancer Institute (NCI) [1]. The HEI-2015 is the most recent iteration and was designed to align with the 2015-2020 DGA. The HEI-2015 comprises of 13 dietary components that sum to a total maximum score of 100 points. The total score is the sum of the scores of nine adequacy components and four moderation components (Supplementary Table S1). Each of the component is a density-based (e.g., amounts per 1,000 kcal) score, except for fatty acids component, which is a ratio of unsaturated to saturated fatty acid. Currently, several methods that have been developed for use with the HEI are available, and the detail and methods associated SAS macros can be accessed on NCI website (https://epi.grants.cancer.gov/hei/hei-methods-and-calculations.html). We used the population ratio method in the current study. The population ratio method [2] is used to calculate the mean intakes of dietary constituents, and scoring standards are applied to arrive at scores at the level of a group of persons. To apply the population ratio method, the intake of the relevant dietary constituents and energy are summed for all individuals in a population to obtain estimates of the population's total intake, and then the ratios of each constituent to energy are computed and scored. The total score is then the sum of the component scores. While this method does not estimate usual intake at the *individual level*, it may be used to estimate usual intake at the *population level*.

## Methods S2.

### *NCI Method*

As previously described, the NCI method is the preferred method for estimating usual dietary intake from 24-hour recalls while accounting for substantial day-to-day variation (also referred to as within-person variability) in individual diets [3,4]. The NCI method requires that at least some individuals in the sample must have multiple dietary recalls (e.g., at least two days of recall) to estimate the within- and between-individual variations [4]. 87.6% of our study population had two dietary recalls. Moreover, the NCI method consists of two steps for usual dietary intake estimation. The first step estimates the probability of consuming a given dietary component and its amount. In this step, reported zero intakes are replaced with one-half of the minimum nonzero value reported in the data set. The specific algorithms used in the NCI method for dietary components that are consumed every day by every individual of the population differ slightly from those used for foods/nutrients that are episodically consumed. For foods/nutrients consumed daily, the amount-only model was used (i.e., MIXTRAN SAS macro). Otherwise, the two-part model (MIXTRAN SAS macro with CORR model) was used. A 10% cutoff for reported zero intake was used to distinguish between ubiquitous or episodic consumption of a given food or nutrient. The second step in the NCI method uses parameters estimated from the first step and a Markov chain Monte Carlo (MCMC) simulation method to model on Box-Cox-transformed intake data as a function of observed fixed-effect covariates, unobserved individual-

level random effects, and within-individual error (i.e., DISTRIB SAS macro) to estimate the distribution of usual intake for a given dietary component [4]. The MCMC method generates a set of representative values whose empirical distribution approximates the assumed distribution of between-individual random effects [5]. Note that as the number of repetitions or the seed number for the random number generator used in the MCMC simulation varies from study to study, differences can occur in the estimation of usual intake even with the same data set. For these analyses, the following covariates were specified for estimating usual intake: 1) an indicator of first- versus second-day 24-hour dietary recall; 2) an indicator of the day of the week when the 24-hour recall was collected, dichotomized as weekend (Friday–Sunday) or weekday (Monday–Thursday); and 3) race/ethnicity. Complete details of the NCI method and the necessary SAS macros needed to perform the Monte Carlo-based estimation of usual intake distributions can be found elsewhere [6].

**Table S1**. Dietary components of Health Eating Index (HEI) 2015 and scoring standards

| HEI-2015 Component | Score Range | Score Standards [a] | |
|---|---|---|---|
| | | Maximum | Minimum |
| **Adequacy components** | | | |
| Total fruits [b] | 0-5 | ≥0.8 cup equivalents/ 1,000 kcal | No |
| Whole fruits [c] | 0-5 | ≥0.4 cup equivalents/ 1,000 kcal | No |
| Total vegetables [d] | 0-5 | ≥ 1.1 cup equivalents/ 1,000 kcal | No |
| Greens and beans [d] | 0-5 | ≥ 1.1 cup equivalents/ 1,000 kcal | No |
| Whole grains | 0-10 | ≥ 1.5 cup equivalents/ 1,000 kcal | No |
| Dairy [e] | 0-10 | ≥ 1.3 cup equivalents/ 1,000 kcal | No |
| Total protein foods [d] | 0-5 | ≥ 2.5 oz equivalents/ 1,000 kcal | No |
| Sea food and plant proteins [d, f] | 0-5 | ≥ 0.8 cup equivalents/ 1,000 kcal | No |
| Fatty acids [g] | 0-10 | (PUFAs + MUFAs)/ SFAs ≥ 2.5 | (PUFAs + MUFAs)/ SFAs ≤ 1.2 |
| **Moderation components** | | | |
| Refined grains | 0-10 | ≤ 1.8 oz equivalents/ 1,000 kcal | ≥ 4.3 oz equivalents/ 1,000 kcal |
| Sodium | 0-10 | ≤ 1.1 gram/ 1,000 kcal | ≥ 2.0 gram/ 1,000 kcal |
| Added sugars | 0-10 | ≤ 6.5% of energy | ≥ 26% of energy |
| Saturated fats | 0-10 | ≤ 8% of energy | ≥ 16% of energy |

[a] Intakes between the minimum and maximum standards are scored proportionately.
[b] Includes 100% fruit juice.
[c] Includes all forms except juice.
[d] Includes legumes (beans and peas).
[e] Includes all milk products, such as fluid milk, yogurt, and cheese, and fortified soy beverages.
[f] Includes seafoods; nuts, seeds, soy products (other than beverages), and legumes (beans and peas).
[g] Ratio of poly- and mono-unsaturated fatty acids (PUFAs and MUFAs) to saturated fatty acids (SFAs).

**Table S2.** Demographics of adults aged ≥20 years by National Health and Nutrition Examination Survey (NHANES) cycle, 2011–2018 *

| Demographics [a] | 2011–2012 (*n* = 4712) | 2013–2014 (*n* = 4939) | 2015–2016 (*n* = 4898) | 2017–2018 (*n* = 4643) | Overall (*n* = 19,192) |
|---|---|---|---|---|---|
| Age (years), n (%) | | | | | 48.23 (0.34) |
| 20–34 | 1258 (27.0) | 1238 (26.9) | 1186 (26.3) | 1015 (26.7) | 4697 (26.7) |
| 35–49 | 1158 (26.4) | 1302 (25.3) | 1219 (25.5) | 1034 (24.0) | 4713 (25.3) |
| 50–64 | 1270 (29.1) | 1296 (28.7) | 1288 (26.5) | 1380 (28.2) | 5234 (28.1) |
| ≥65 | 1026 (17.5) | 1103 (19.1) | 1205 (21.7) | 1214 (21.1) | 4548 (19.9) |
| Gender, n (%) | | | | | |
| Male | 2380 (48.9) | 2411 (49.1) | 2406 (49.4) | 2301 (48.7) | 9498 (49.0) |
| Female | 2332 (51.1) | 2528 (50.9) | 2492 (50.6) | 2342 (51.3) | 9694 (51.0) |
| Race/Ethnicity, n (%) | | | | | |
| Non-Hispanic white | 1810 (67.1) | 2188 (65.6) | 1684 (65.3) | 1664 (63.0) | 7346 (65.2) |
| Non-Hispanic black | 1246 (11.4) | 989 (11.3) | 1034 (10.7) | 1102 (11.6) | 4371 (11.3) |
| Hispanics | 915 (14.2) | 1106 (14.8) | 1497 (14.4) | 1030 (15.1) | 4548 (14.6) |
| Non-Hispanic Asian | 600 (5.2) | 508 (5.3) | 507 (5.7) | 613 (5.8) | 2228 (5.5) |
| Other race [b] | 141 (2.1) | 148 (3.0) | 179 (3.9) | 234 (4.4) | 699 (3.4) |
| Education, n (%) | | | | | |
| Less than high school | 1088 (16.0) | 1008 (13.8) | 1123 (13.0) | 869 (9.5) | 4088 (13.0) |
| High school | 991 (20.4) | 1124 (21.2) | 1086 (21.5) | 1127 (27.2) | 4328 (22.6) |
| More than high school | 2630 (63.6) | 2804 (65.0) | 2687 (65.5) | 2639 (62.3) | 10760 (64.3) |
| PIR, n (%) [c] | | | | | |
| <1.33 | 1576 (25.3) | 1568 (24.5) | 1417 (20.5) | 1158 (19.6) | 5719 (22.4) |
| 1.33–3.29 | 1348 (30.1) | 1445 (30.8) | 1637 (33.9) | 1541 (31.1) | 5971 (31.5) |
| ≥3.30 | 1422 (44.7) | 1572 (44.7) | 1370 (45.6) | 1398 (49.4) | 5762 (46.1) |
| BMI (kg/m$^2$), n (%) | | | | | |
| <25.0 | 1456 (31.6) | 1452 (29.5) | 1285 (26.9) | 1175 (26.0) | 5368 (28.5) |
| 25.0–<30.0 | 1491 (33.3) | 1578 (32.7) | 1570 (32.9) | 1465 (30.1) | 6104 (32.2) |
| ≥30.0 | 1705 (35.1) | 1872 (37.8) | 1996 (40.2) | 1953 (43.9) | 7526 (39.3) |

* The numbers may not sum to the total number of participants due to missing data (n = 16 without education data; n = 1740 without income data; n = 194 without BMI data). [a] Values are presented as unweighted frequency (weighted percentages); [b] Other race: multiple races [c] PIR: poverty-income ratio.

**Table S3**. Trends in estimated HEI-2015 among adults aged ≥20 years, by NHANES cycles from 2011 to 2018

| HEI-2015 scores | Scoring | | | Survey-weighted mean score (95% CI) | | | | Differences 2017-2018 *vs.* 2011-2012 (95% CI) | *p* for trend [a] |
|---|---|---|---|---|---|---|---|---|---|
| | Range | Max | Min | 2011–2012 (*n* = 4313) | 2013–2014 (*n* = 4559) | 2015–2016 (*n* = 4394) | 2017–2018 (*n* = 4058) | | |
| **Total** | 0-100 | 100 | 0 | 55.01 (54.09, 55.95) | 54.18 (53.46, 54.91) | 53.91 (52.46, 55.36) | 52.65 (51.12, 54.19) | -2.36 (-4.16, -0.57) | 0.011 |
| **Adequacy components** | | | | | | | | | |
| Total fruits [b] | 0-5 | ≥0.8 c /1000 kcal | No | 2.47 (2.38, 2.58) | 2.31 (2.21, 2.41) | 2.27 (2.11, 2.44) | 2.19 (2.01, 2.37) | -0.29 (-0.50, -0.08) | 0.007 |
| Whole fruits [c] | 0-5 | ≥0.4 c /1000 kcal | No | 2.59 (2.45, 2.74) | 2.56 (2.43, 2.68) | 2.47 (2.27, 2.68) | 2.44 (2.21, 2.68) | -0.15 (-0.43, 0.13) | 0.233 |
| Total vegetables [d] | 0-5 | ≥ 1.1 c /1000 kcal | No | 3.37 (3.29, 3.45) | 3.26 (3.17, 3.35) | 3.29 (3.22, 3.36) | 3.22 (3.10, 3.34) | -0.14 (-0.29, 0.00) | 0.081 |
| Greens and beans [d] | 0-5 | ≥ 1.1 c /1000 kcal | No | 2.04 (1.92, 2.17) | 2.08 (1.98, 2.18) | 2.16 (2.01, 2.31) | 2.00 (1.84, 2.16) | -0.04 (-0.25, 0.16) | 0.863 |
| Whole grains | 0-10 | ≥ 1.5 c /1000 kcal | No | 3.06 (2.86, 3.27) | 2.95 (2.84, 3.06) | 3.01 (2.79, 3.23) | 2.64 (2.38, 2.90) | -0.42 (-0.75, -0.09) | 0.020 |
| Dairy [e] | 0-10 | ≥ 1.3 c /1000 kcal | No | 5.39 (5.22, 5.56) | 5.49 (5.32, 5.67) | 5.25 (4.99, 5.51) | 4.93 (4.76, 5.10) | -0.46 (-0.6, -0.22) | <0.001 |
| Total protein foods [d] | 0-5 | ≥ 2.5 c /1000 kcal | No | 4.48 (4.42, 4.54) | 4.50 (4.45, 4.54) | 4.54 (4.50, 4.58) | 4.53 (4.46, 4.59) | 0.05 (-0.04, 0.14) | 0.174 |
| Sea food and plant proteins [d, f] | 0-5 | ≥ 0.8 c /1000 kcal | No | 2.90 (2.80, 3.00) | 2.97 (2.87, 3.07) | 3.04 (2.89, 3.20) | 2.95 (2.77, 3.12) | 0.04 (-0.16, 0.25) | 0.537 |
| Fatty acids [g] | 0-10 | Ratio ≥ 2.5 | ≤ 1.2 | 5.29 (5.12, 5.46) | 4.95 (4.79, 5.11) | 4.88 (4.67, 5.10) | 4.85 (4.64, 5.06) | -0.44 (-0.71, -0.16) | 0.002 |
| **Moderation components** | | | | | | | | | |
| Refined grains | 0-10 | ≤ 1.8 c /1000 kcal | ≥ 4.3 c /1000 kcal | 6.18 (5.95, 6.40) | 6.27 (6.09, 6.44) | 6.48 (6.26, 6.70) | 6.30 (6.08, 6.52) | 0.12 (-0.19, 0.43) | 0.256 |
| Sodium | 0-10 | ≤ 1.1 g /1000 kcal | ≥ 2.0 g /1000 kcal | 4.00 (3.93, 4.07) | 3.97 (3.74, 4.20) | 3.88 (3.69, 4.08) | 4.28 (4.09, 4.47) | 0.28 (0.08, 0.49) | 0.030 |
| Added sugars | 0-10 | ≤ 6.5% of energy | ≥ 26% of energy | 6.83 (6.62, 7.05) | 6.87 (6.65, 7.09) | 7.09 (6.86, 7.32) | 7.07 (6.82, 7.31) | 0.23 (-0.10, 0.56) | 0.084 |
| Saturated fats | 0-10 | ≤ 8% of energy | ≥ 16% of energy | 6.41 (6.19, 6.63) | 6.01 (5.83, 6.18) | 5.53 (5.32, 5.75) | 5.26 (5.05, 5.47) | -1.15 (-1.45, -0.85) | <0.001 |

[a] *p* value from trend tests by modeling survey period as a continuous variable. [b] Includes 100% fruit juice. [c] Includes all forms except juice. [d] Includes legumes (beans and peas). [e] Includes all milk products, such as fluid milk, yogurt, and cheese, and fortified soy beverages. [f] Includes seafood, nuts, seeds, soy products (other than beverages), and legumes (beans and peas). [g] Ratio of PUFAs and MUFAs to SFAs ((PUFAs+MUFAs)/ SFAs). 1 c. = 1 cup equivalents

**Table S4.** Trends in estimated HEI-2015 by race/ethnicity among adults aged ≥20 years, NHANES 2011-2018

| HEI-2015 scores | Survey-weighted mean score (95% CI) | | | | Differences 2017-2018 *vs.* 2011-2012 (95% CI) | *p* for trend [a] |
|---|---|---|---|---|---|---|
| | 2011–2012 (*n* = 4313) | 2013–2014 (*n* = 4559) | 2015–2016 (*n* = 4394) | 2017–2018 (*n* = 4058) | | |
| **Total** | | | | | | |
| NH white | 55.36 (54.19, 56.53) | 54.03 (53.21, 54.86) | 54.15 (52.50, 55.80) | 52.14 (50.30, 53.98) | -3.22 (-5.40, -1.04) | 0.007 |
| NH black | 52.48 (50.27, 54.70) | 51.75 (50.57, 50.92) | 50.63 (48.67, 52.59) | 50.82 (49.04, 52.59) | -1.67 (-4.50, 1.17) | 0.176 |
| Hispanic | 54.06 (52.91, 55.21) | 54.58 (52.64, 56.52) | 52.75 (51.28, 54.21) | 54.04 (52.10, 55.99) | -0.02 (-2.28, 2.25) | 0.625 |
| NH Asian | 59.85 (58,09, 61.61) | 61.16 (59.54, 62.79) | 60.52 (58.89, 62.16) | 59.85 (57.36, 62.33) | -0.01 (-3.05, 3.04) | 0.855 |
| Other race | 52.83 (48.68, 56.38) | 52.36 (48.10, 56.63) | 53.57 (50.12, 57.03) | 50.60 (46.84, 54.35) | -1.93 (-7.31, 3.44) | 0.498 |
| **Adequacy components** | | | | | | |
| Total fruits [b] | | | | | | |
| NH white | 2.45 (2.31, 2.59) | 2.23 (2.09, 2.37) | 2.20 (1.97, 2.42) | 2.06 (1.83, 2.30) | -0.39 (-0.66, -0.12) | 0.007 |
| NH black | 2.36 (2.09, 2.63) | 2.26 (2.10, 2.42) | 2.20 (1.95, 2.44) | 2.07 (1.75, 2.38) | -0.29 (-0.71, 0.12) | 0.145 |
| Hispanic | 2.56 (2.44, 2.68) | 2.58 (2.41, 2.75) | 2.55 (2.36, 2.73) | 2.57 (2.34, 2.80) | 0.01 (-0.25, 0.28) | 0.979 |
| NH Asian | 2.98 (2.80, 3.16) | 2.94 (2.70, 3.18) | 2.92 (2.69, 3.14) | 3.01 (2.77, 3.25) | 0.03 (-0.27, 0.32) | 0.885 |
| Other race | 2.15 (1.73, 2.57) | 1.90 (1.29, 2.51) | 1.83 (1.38, 2.28) | 1.87 (1.45, 2.28) | -0.29 (-0.88, 0.31) | 0.461 |
| Whole fruits [c] | | | | | | |
| NH white | 2.65 (2.48, 2.83) | 2.57 (2.40, 2.74) | 2.47 (2.24, 2.71) | 2.42 (2.10, 2.74) | -0.23 (-0.60, 0.14) | 0.173 |
| NH black | 1.97 (1.61, 2.32) | 2.05 (1.82, 2.28) | 1.98 (1.71, 2.25) | 1.89 (1.55, 2.22) | -0.08 (-0.57, 0.41) | 0.675 |
| Hispanic | 2.61 (2.44, 2.79) | 2.73 (2.53, 2.92) | 2.56 (2.36, 2.77) | 2.70 (2.40, 3.00) | 0.09 (-0.26, 0.43) | 0.857 |
| NH Asian | 3.34 (3.09, 3.58) | 3.26 (2.98, 3.54) | 3.32 (3.09, 3.55) | 3.36 (3.11, 3.62) | 0.03 (-0.33, 0.39) | 0.780 |
| Other race | 2.14 (1.74, 2.55) | 2.13 (1.45, 2.81) | 2.26 (1.74, 2.77) | 2.13 (1.72, 2.53) | -0.02 (-0.59, 0.55) | 0.987 |
| Total vegetables [d] | | | | | | |
| NH white | 3.38 (3.29, 3.48) | 3.25 (3.13, 3.36) | 3.30 (3.24, 3.36) | 3.22 (3.06, 3.38) | -0.17 (-0.35, 0.02) | 0.124 |
| NH black | 3.01 (2.88, 3.14) | 2.99 (2.85, 3.13) | 2.99 (2.87, 3.12) | 2.88 (2.74, 3.01) | -0.13 (-0.32, 0.05) | 0.167 |
| Hispanic | 3.44 (3.30, 3.57) | 3.37 (3.26, 3.48) | 3.25 (3.14, 3.37) | 3.38 (3.22, 3.54) | -0.06 (-0.27, 0.16) | 0.438 |
| NH Asian | 3.76 (2.83, 3.95) | 3.82 (3.63, 4.00) | 3.88 (3.71, 4.04) | 3.64 (3.42, 3.87) | -0.12 (-0.41, 0.17) | 0.467 |
| Other race | 3.32 (2.83, 3.82) | 2.98 (2.73, 3.23) | 3.19 (2.74, 3.64) | 3.13 (2.92, 3.33) | -0.19 (-0.72, 0.35) | 0.809 |
| Greens and beans [d] | | | | | | |

| | | | | | | |
|---|---|---|---|---|---|---|
| NH white | 1.92 (1.78, 2.06) | 1.98 (1.85, 2.12) | 2.07 (1.86, 2.27) | 1.83 (1.62, 2.04) | -0.08 (-0.34, 0.17) | 0.691 |
| NH black | 1.76 (1.56, 1.97) | 1.71 (1.57, 1.84) | 1.74 (1.48, 2.00) | 1.69 (1.57, 1.81) | -0.08 (-0.31, 0.16) | 0.589 |
| Hispanic | 2.58 (2.31, 2.85) | 2.54 (2.43, 2.65) | 2.47 (2.31, 2.63) | 2.61 (2.40, 2.82) | 0.03 (-0.31, 0.37) | 0.957 |
| NH Asian | 2.93 (2.62, 3.24) | 2.86 (2.52, 3.20) | 3.27 (3.02, 3.51) | 2.92 (2.47, 3.36) | -0.01 (-0.55, 0.53) | 0.687 |
| Other race | 1.78 (1.03, 2.53) | 1.92 (1.26, 2.58) | 2.22 (1.61, 2.84) | 1.87 (1.53, 2.21) | 0.09 (-0.73, 0.92) | 0.800 |
| Whole grains | | | | | | |
| NH white | 3.28 (3.07, 3.49) | 3.09 (2.95, 3.24) | 3.21 (2.97, 3.47) | 2.71 (2.37, 3.05) | -0.57 (-0.97, -0.18) | 0.012 |
| NH black | 2.47 (2.06, 2.89) | 2.55 (2.31, 2.78) | 2.26 (1.95, 2.58) | 2.20 (1.98, 2.41) | -0.28 (-0.75, 0.19) | 0.139 |
| Hispanic | 2.28 (2.01, 2.54) | 2.31 (2.02, 2.60) | 2.31 (2.10, 2.53) | 2.23 (1.90, 2.57) | -0.04 (-0.47, 0.38) | 0.844 |
| NH Asian | 3.65 (3.11, 4.20) | 3.90 (3.27, 4.52) | 3.86 (3.49, 4.23) | 3.94 (3.57, 4.32) | 0.29 (-0.37, 0.95) | 0.435 |
| Other race | 3.17 (2.71, 3.63) | 2.85 (1.88, 3.82) | 2.89 (2.25, 3.54) | 2.47 (1.74, 3.20) | -0.70 (-1.57, 0.16) | 0.203 |
| Dairy [e] | | | | | | |
| NH white | 5.75 (5.55, 5.94) | 5.84 (5.60, 6.07) | 5.67 (5.49, 5.85) | 5.24 (5.03, 5.44) | -0.51 (-0.80, -0.22) | <0.001 |
| NH black | 3.98 (3.73, 4.23) | 4.09 (3.86, 4.32) | 3.75 (3.36, 4.13) | 3.83 (3.64, 4.02) | -0.15 (-0.46, 0.16) | 0.142 |
| Hispanic | 5.42 (5.16, 5.68) | 5.47 (5.15, 5.78) | 5.07 (4.76, 5.38) | 4.85 (4.52, 5.17) | -0.57 (-0.98, -0.15) | 0.003 |
| NH Asian | 3.96 (3.64, 4.28) | 4.37 (4.08, 4.66) | 4.08 (3.60, 4.55) | 4.29 (3.80, 4.78) | 0.34 (-0.25, 0.92) | 0.459 |
| Other race | 4.95 (4.40, 5.50) | 5.34 (4.35, 6.32) | 4.66 (3.91, 5.42) | 4.62 (4.08, 5.16) | -0.34 (-1.10, 0.43) | 0.189 |
| Total protein foods [d] | | | | | | |
| NH white | 4.41 (4.34, 4.48) | 4.46 (4.39, 4.52) | 4.50 (4.44, 4.56) | 4.51 (4.41, 4.60) | 0.10 (-0.02, 0.22) | 0.065 |
| NH black | 4.66 (4.60, 4.71) | 4.58 (4.49, 4.66) | 4.58 (4.50, 4.66) | 4.56 (4.48, 4.64) | -0.10 (-0.19, 0.001) | 0.081 |
| Hispanic | 4.63 (4.57, 4.70) | 4.57 (4.46, 4.68) | 4.62 (4.43, 4.79) | 4.62 (4.54, 4.71) | -0.01 (-0.12, 0.09) | 0.940 |
| NH Asian | 4.60 (4.49, 4.72) | 4.70 (4.61, 4.80) | 4.61 (4.43, 4.79) | 4.49 (4.33, 4.69) | -0.12 (-0.31, 0.08) | 0.148 |
| Other race | 4.44 (4.31, 4.56) | 4.34 (4.16, 4.51) | 4.74 (4.64, 4.79) | 4.45 (4.21, 4.69) | 0.01 (-0.26, 0.28) | 0.473 |
| Seafood and plant proteins [d, f] | | | | | | |
| NH white | 2.84 (2.71, 2.98) | 2.90 (2.80, 3.01) | 3.07 (2.88, 3.26) | 2.92 (2.72, 3.1.) | 0.08 (-0.17, 0.33) | 0.299 |
| NH black | 2.66 (2.33, 2.99) | 2.70 (2.48, 2.93) | 2.42 (2.18, 2.65) | 2.58 (2.35, 2.82) | -0.08 (-0.48, 0.33) | 0.444 |
| Hispanic | 3.13 (2.93, 3.32) | 3.22 (3.03, 3.41) | 3.02 (2.87, 3.17) | 3.29 (3.01, 3.56) | 0.16 (-0.18, 0.50) | 0.571 |
| NH Asian | 3.74 (3.56, 3.92) | 3.86 (3.67, 4.04) | 3.90 (3.63, 4.18) | 3.50 (3.15, 3.86) | -0.23 (-0.63, 0.17) | 0.258 |
| Other race | 2.55 (1.94, 3.13) | 2.73 (1.99, 3.47) | 3.14 (2.61, 3.66) | 2.36 (1.65, 3.06) | -0.19 (-1.11, 0.72) | 0.661 |

| | | | | | | |
|---|---|---|---|---|---|---|
| Fatty acids [g] | | | | | | |
| NH white | 5.04 (4.81, 5.27) | 4.74 (4.50, 4.98) | 4.58 (4.32, 4.84) | 4.54 (4.26, 4.82) | -0.50 (-0.87, -0.14) | 0.005 |
| NH black | 6.07 (5.77, 6.36) | 5.39 (4.99, 5.80) | 5.70 (5.29, 6.10) | 5.81 (5.55, 6.07) | -0.25 (-0.65, 0.14) | 0.545 |
| Hispanic | 5.28 (5.04, 5.52) | 4.86 (4.34, 5.39) | 4.89 (4.56, 5.22) | 4.90 (4.54, 5.23) | -0.38 (-0.81, 0.06) | 0.160 |
| NH Asian | 6.63 (6.29, 6.97) | 6.80 (6.39, 7.21) | 6.45 (6.14, 6.76) | 6.03 (5.46, 6.60) | -0.60 (-1.26, 0.07) | 0.046 |
| Other race | 5.64 (4.58, 6.71) | 5.08 (4.20, 5.95) | 5.43 (4.71, 6.15) | 5.03 (4.08, 5.98) | -0.61 (-2.04, 0.81) | 0.547 |
| **Moderation components** | | | | | | |
| Refined grains | | | | | | |
| NH white | 6.47 (6.22, 6.73) | 6.49 (6.27, 6.71) | 6.88 (6.26, 6.70) | 6.58 (6.32, 6.83) | 0.11 (-0.25, 0.47) | 0.208 |
| NH black | 6.87 (6.52, 7321) | 6.86 (6.58, 7.14) | 6.77 (6.45, 7.09) | 6.63 (6.25, 7.01) | -0.24 (-0.75, 0.28) | 0.320 |
| Hispanic | 4.69 (4.20, 5.18) | 5.15 (4.66, 5.65) | 4.67 (4.10, 5.23) | 5.21 (4.82, 5.59) | 0.52 (-0.11, 1.14) | 0.288 |
| NH Asian | 5.14 (4.79, 5.48) | 5.40 (4.89, 5.92) | 5.42 (5.05, 5.79) | 5.37 (5.01, 5.74) | 0.24 (-0.27, 0.74) | 0.403 |
| Other race | 5.72 (4.75, 6.69) | 6.19 (5.05, 7.33) | 7.13 (6.48, 7.78) | 6.42 (5.61, 7.23) | 0.70 (-0.57, 1.97) | 0.252 |
| Sodium | | | | | | |
| NH white | 4.11 (4.01, 4.21) | 3.93 (3.65, 4.21) | 3.97 (3.70, 4.24) | 4.28 (4.02, 4.54) | 0.17 (-0.11, 0.45) | 0.251 |
| NH black | 4.09 (3.87, 4.31) | 4.25 (4.02, 4.47) | 4.04 (3.74, 4.33) | 4.34 (4.07, 4.62) | 0.26 (-0.09, 0.61) | 0.303 |
| Hispanic | 3.87 (3.53, 4.20) | 4.26 (3.92, 4.61) | 4.05 (3.76, 4.34) | 4.36 (3.99, 4.73) | 0.49 (-0.01, 0.99) | 0.121 |
| NH Asian | 2.74 (2.43, 3.05) | 2.73 (2.34, 3.12) | 2.46 (2.11, 2.80) | 3.66 (3.48, 3.85) | 0.92 (0.56, 1.28) | <0.001 |
| Other race | 3.79 (3.29, 4.28) | 4.49 (3.53, 5.45) | 3.48 (2.88, 4.08) | 4.59 (3.91, 5.26) | 0.80 (-0.04, 1.64) | 0.310 |
| Added sugar | | | | | | |
| NH white | 6.89 (6.61, 7.16) | 6.88 (6.58, 7.19) | 7.06 (6.78, 7.35) | 7.02 (6.70, 7.35) | 0.14 (-0.29, 0.56) | 0.378 |
| NH black | 5.90 (5.63, 6.17) | 6.04 (5.71, 6.37) | 6.21 (5.80, 6.61) | 6.46 (6.19, 6.74) | 0.56 (0.17, 0.95) | 0.005 |
| Hispanic | 6.96 (6.64, 7.28) | 7.02 (6.71, 7.32) | 7.14 (6.90, 7.38) | 7.37 (7.02, 7.72) | 0.41 (-0.07, 0.88) | 0.074 |
| NH Asian | 8.28 (7.92, 8.64) | 8.56 (8.24, 8.88) | 8.76 (8.38, 9.13) | 8.54 (8.32, 8.77) | 0.27 (-0.16, 0.69) | 0.160 |
| Other race | 5.83 (4.86, 6.80) | 6.02 (5.06, 6.99) | 7.30 (6.69, 7.90) | 6.25 (5.80, 6.70) | 0.42 (-0.65, 1.49) | 0.272 |
| Saturated fat | | | | | | |
| NH white | 6.16 (5.88, 6.63) | 5.67 (5.44, 5.91) | 5.16 (4.88, 5.43) | 4.80 (4.54, 5.07) | -1.36 (-1.75, -0.97) | <0.001 |
| NH black | 6.70 (6.45, 6.94) | 6.28 (5.85, 6.71) | 6.01 (5.56, 6.46) | 5.88 (5.55, 6.21) | -0.82 (-1.23, -0.40) | <0.001 |
| Hispanic | 6.62 (6.32, 6.91) | 6.50 (6.20, 6.81) | 6.15 (5.86, 6.44) | 5.95 (5.64, 6.26) | -0.67 (-1.09, -0.24) | 0.001 |
| NH Asian | 8.11 (7.78, 8.44) | 7.97 (7.69, 8.25) | 7.60 (7.31, 7.90) | 7.08 (6.57, 7.58) | -1.03 (-1.63, -0.43) | 0.001 |

| | | | | | | |
|---|---|---|---|---|---|---|
| Other race | 7.04 (6.38, 7.70) | 6.40 (5.79, 7.00) | 5.31 (4.73, 5.89) | 5.42 (4.42, 6.39) | -1.62 (-2.79, -0.45) | 0.006 |

[a] *p* value from trend tests by modeling survey period as a continuous variable. [b] Includes 100% fruit juice. [c] Includes all forms except juice. [d] Includes legumes (beans and peas). [e] Includes all milk products, such as fluid milk, yogurt, and cheese, and fortified soy beverages. [f] Includes seafood, nuts, seeds, soy products (other than beverages), and legumes (beans and peas). [g] Ratio of PUFAs and MUFAs to SFAs ((PUFAs+MUFAs)/ SFAs). 1 c. = 1 cup equivalents

**Table S5**. Trends in estimated mean consumption of selected food groups and nutrients by race/ethnicity among adults aged ≥20 years, NHANES 2011-2018

| Foods/Nutrients | Survey-weighted mean score (95% CI) | | | | Differences 2017-2018 *vs.* 2011-2012 (95% CI) | *p* for trend [a] |
|---|---|---|---|---|---|---|
| | 2011–2012 (*n* = 4313) | 2013–2014 (*n* = 4559) | 2015–2016 (*n* = 4394) | 2017–2018 (*n* = 4058) | | |
| **Food density (per 2000 kcal per day)** | | | | | | |
| Total fruits (servings) | | | | | | |
| NH white | 0.99 (0.88, 1.10) | 0.92 (0.83, 1.01) | 0.91 (0.79, 1.02) | 0.81 (0.71, 0.91) | -0.18 (-0.31, -0.05) | 0.001 |
| NH black | 0.96 (0.83, 1.09) | 0.86 (0.76, 0.97) | 0.92 (0.80, 1.04) | 0.88 (0.68, 1.09) | -0.08 (-0.30, 0.15) | 0.830 |
| Hispanic | 1.06 (0.97, 1.16) | 1.12 (0.99, 1.25) | 1.13 (1.04, 1.22) | 1.19 (1.05, 1.32) | 0.12 (-0.03, 0.27) | 0.382 |
| NH Asian | 1.38 (1.25, 1.51) | 1.40 (1.26, 1.55) | 1.44 (1.28, 1.60) | 1.45 (1.25, 1.64) | 0.07 (-0.15, 0.28) | 0.708 |
| Other race | 0.92 (0.79, 1.06) | 0.79 (0.45, 1.14) | 0.72 (0.55, 0.89) | 0.79 (0.62, 0.95) | -0.14 (-0.33, 0.06) | 0.107 |
| Intact/whole fruit | | | | | | |
| NH white | 0.74 (0.65, 0.82) | 0.71 (0.62, 0.79) | 0.71 (0.61, 0.82) | 0.66 (0.56, 0.75) | -0.08 (-0.20, 0.03) | 0.022 |
| NH black | 0.53 (0.42, 0.63) | 0.52 (0.44, 0.61) | 0.57 (0.46, 0.69) | 0.58 (0.42, 0.74) | 0.05 (-0.13, 0.23) | 0.603 |
| Hispanic | 0.73 (0.63, 0.83) | 0.82 (0.70, 0.93) | 0.80 (0.71, 0.90) | 0.90 (0.76, 1.04) | 0.17 (0.02, 0.33) | 0.156 |
| NH Asian | 1.07 (0.96, 1.19) | 1.17 (1.03, 1.30) | 1.22 (1.07, 1.37) | 1.22 (1.03, 1.41) | 0.15 (-0.06, 0.35) | 0.206 |
| Other race | 0.56 (0.37, 0.75) | 0.62 (0.30, 0.93) | 0.57 (0.44, 0.71) | 0.64 (0.49, 0.79) | 0.08 (-0.14, 0.31) | 0.634 |
| 100% fruit juices | | | | | | |
| NH white | 0.25 (0.19, 0.31) | 0.21 (0.19, 0.24) | 0.21 (0.18, 0.24) | 0.16 (0.14, 0.19) | -0.09 (-0.14, -0.03) | <0.001 |
| NH black | 0.48 (0.41, 0.54) | 0.38 (0.32, 0.45) | 0.41 (0.33, 0.49) | 0.34 (0.25, 0.42) | -0.14 (-0.24, -0.04) | 0.069 |
| Hispanic | 0.39 (0.31, 0.46) | 0.37 (0.31, 0.42) | 0.38 (0.30, 0.46) | 0.32 (0.28, 0.37) | -0.06 (-0.14, 0.02) | 0.143 |

| | | | | | | |
|---|---|---|---|---|---|---|
| NH Asian | 0.32 (0.25, 0.39) | 0.25 (0.18, 0.31) | 0.27 (0.19, 0.34) | 0.18 (0.13, 0.23) | -0.14 (-0.22, -0.06) | 0.001 |
| Other race | 0.35 (0.16, 0.54) | 0.22 (0.09, 0.35) | 0.17 (0.10, 0.23) | 0.16 (0.08, 0.24) | -0.19 (-0.38, 0.005) | 0.001 |
| Total vegetables (servings) | | | | | | |
| NH white | 1.70 (1.59, 1.82) | 1.60 (1.50, 1.70) | 1.68 (1.57, 1.79) | 1.61 (1.49, 1.74) | -0.09 (-0.24, 0.07) | 0.285 |
| NH black | 1.40 (1.29, 1.51) | 1.34 (1.25, 1.43) | 1.44 (1.37, 1.50) | 1.40 (1.29, 1.51) | 0.005 (-0.14, 0.15) | 0.487 |
| Hispanic | 1.54 (1.44, 1.65) | 1.49 (1.42, 1.57) | 1.47 (1.37, 1.58) | 1.58 (1.47, 1.69) | 0.04 (-0.10, 0.17) | 0.898 |
| NH Asian | 1.90 (1.78, 2.03) | 1.97 (1.78, 2.16) | 2.02 (1.87, 2.16) | 1.93 (1.71, 2.15) | 0.03 (-0.21, 0.26) | 0.744 |
| Other race | 1.51 (1.19, 1.83) | 1.26 (1.01, 1.50) | 1.51 (1.21, 1.80) | 1.53 (1.33, 1.74) | 0.03 (-0.32, 0.38) | 0.394 |
| Total grains (servings) | | | | | | |
| NH white | 6.10 (5.90, 6.30) | 6.02 (5.89, 6.15) | 5.77 (5.68, 5.86) | 5.93 (5.74, 6.13) | -0.17 (-0.43, 0.09) | 0.014 |
| NH black | 5.87 (5.67, 6.08) | 5.83 (5.66, 5.99) | 5.80 (5.52, 6.07) | 5.75 (5.49, 6.01) | -0.12 (-0.43, 0.18) | 0.269 |
| Hispanic | 6.97 (6.68, 7.26) | 6.98 (6.61, 7.34) | 7.10 (6.73, 7.48) | 6.80 (6.44, 7.16) | -0.17 (-0.60, 0.26) | 0.607 |
| NH Asian | 7.39 (7.18, 7.61) | 7.20 (6.88, 7.52) | 7.42 (7.06, 7.78) | 7.45 (7.02, 7.88) | 0.05 (-0.39, 0.49) | 0.524 |
| Other race | 6.79 (6.33, 7.25) | 6.46 (5.79, 7.14) | 5.74 (5.29, 6.20) | 5.84 (5.23, 6.50) | -0.94 (-1.64, -0.24) | 0.016 |
| Whole grains | | | | | | |
| NH white | 1.05 (0.94, 1.16) | 0.97 (0.88, 1.05) | 1.00 (0.92, 1.07) | 0.82 (0.69, 0.96) | -0.23 (-0.39, -0.06) | 0.011 |
| NH black | 0.81 (0.65, 0.97) | 0.79 (0.72, 0.86) | 0.76 (0.63, 0.88) | 0.67 (0.61, 0.74) | -0.14 (-0.29, 0.02) | 0.080 |
| Hispanic | 0.74 (0.60, 0.88) | 0.73 (0.63, 0.83) | 0.70 (0.61, 0.79) | 0.65 (0.58, 0.72) | -0.09 (-0.24, 0.05) | 0.217 |
| NH Asian | 1.24 (1.02, 1.47) | 1.29 (1.02, 1.56) | 1.29 (1.11, 1.47) | 1.31 (1.13, 1.49) | 0.07 (-0.20, 0.33) | 0.654 |
| Other race | 1.08 (0.86, 1.30) | 0.90 (0.57, 1.22) | 0.56 (0.64, 1.08) | 0.72 (0.46, 0.99) | -0.35 (-0.67, -0.03) | 0.062 |
| Refined grains | | | | | | |

| | | | | | | |
|---|---|---|---|---|---|---|
| NH white | 5.07 (4.90, 5.25) | 5.07 (4.90, 5.24) | 4.81 (4.69, 4.93) | 5.11 (4.95, 5.26) | 0.04 (-0.18, 0.25) | 0.357 |
| NH black | 5.09 (4.84, 5.34) | 5.04 (4.86, 5.22) | 5.01 (4.75, 5.27) | 5.07 (4.76, 5.39) | -0.01 (-0.38, 0.36) | 0.937 |
| Hispanic | 6.25 (5.94, 6.57) | 6.26 (5.91, 6.62) | 6.39 (5.98, 6.80) | 6.12 (5.74, 6.49) | -0.13 (-0.58, 0.32) | 0.918 |
| NH Asian | 6.09 (5.82, 6.37) | 5.80 (5.52, 6.09) | 5.98 (5.67, 6.28) | 6.05 (5.69, 6.41) | -0.04 (-0.46, 0.37) | 0.658 |
| Other race | 5.79 (5.35, 6.23) | 5.62 (4.93, 6.30) | 4.93 (4.57, 5.30) | 5.17 (4.55, 5.80) | -0.61 (-1.31, 0.09) | 0.089 |
| Legumes (servings) | | | | | | |
| NH white | 0.10 (0.08, 0.12) | 0.08 (0.07, 0.10) | 0.10 (0.07, 0.12) | 0.08 (0.06, 0.10) | -0.02 (-0.04, 0.01) | 0.439 |
| NH black | 0.08 (0.07, 0.09) | 0.09 (0.07, 0.11) | 0.08 (0.05, 0.11) | 0.08 (0.06, 0.09) | -0.01 (-0.03, 0.01) | 0.275 |
| Hispanic | 0.23 (0.20, 0.26) | 0.23 (0.18, 0.28) | 0.23 (0.20, 0.26) | 0.22 (0.17, 0.28) | -0.004 (-0.06, 0.05) | 0.831 |
| NH Asian | 0.15 (0.12, 0.18) | 0.14 (0.11, 0.18) | 0.13 (0.09, 0.16) | 0.17 (0.05, 0.28) | 0.02 (-0.09, 0.13) | 0.803 |
| Other race | 0.10 (0.02, 0.19) | 0.11 (0.04, 0.19) | 0.08 (0.03, 0.12) | 0.07 (0.02, 0.12) | -0.03 (-0.13, 0.06) | 0.470 |
| Soy products (servings) | | | | | | |
| NH white | 0.07 (0.05, 0.09) | 0.08 (0.06, 0.10) | 0.15 (0.11, 0.19) | 0.11 (0.07, 0.15) | 0.04 (-0.005, 0.08) | 0.013 |
| NH black | 0.05 (0.03, 0.06) | 0.04 (0.02, 0.05) | 0.04 (0.02, 0.06) | 0.06 (0.04, 0.08) | 0.01 (-0.01, 0.04) | 0.465 |
| Hispanic | 0.05 (0.02, 0.09) | 0.05 (0.02, 0.08) | 0.05 (0.02, 0.09) | 0.12 (0.06, 0.17) | 0.06 (0.003, 0.12) | 0.095 |
| NH Asian | 0.18 (0.09, 0.27) | 0.27 (0.17, 0.37) | 0.23 (0.16, 0.31) | 0.16 (0.08, 0.25) | -0.02 (-0.13, 0.09) | 0.311 |
| Other race | 0.07 (0.03, 0.12) | 0.05 (0.01, 0.08) | 0.08 (-0.01, 0.18) | 0.19 (0.07, 0.32) | 0.12 (-0.002, 0.24) | 0.006 |
| Total meat (servings) | | | | | | |
| NH white | 4.32 (4.08, 4.55) | 4.62 (4.32, 4.91) | 4.53 (4.23, 4.83) | 4.49 (4.24, 4.74) | 0.17 (-0.14, 0.48) | 0.741 |
| NH black | 5.57 (5.41, 5.73) | 5.56 (5.26, 5.85) | 5.51 (5.24, 5.79) | 5.24 (5.06, 5.42) | -0.33 (-0.55, -0.10) | 0.009 |
| Hispanic | 4.98 (4.76, 5.20) | 4.89 (4.58, 5.21) | 4.97 (4.74, 5.21) | 4.68 (4.45, 4.90) | -0.30 (-0.59, -0.01) | 0.139 |

| | | | | | | |
|---|---|---|---|---|---|---|
| NH Asian | 5.02 (4.69, 5.36) | 5.10 (4.71, 5.48) | 4.95 (4.40, 5.50) | 4.60 (3.81, 5.39) | -0.43 (-1.21, 0.36) | 0.211 |
| Other race | 4.34 (3.78, 4.90) | 4.47 (3.92, 5.02) | 5.34 (4.80, 5.88) | 4.49 (4.07, 4.91) | 0.15 (-0.49, 0.79) | 0.478 |
| Unprocessed red meat (servings) | | | | | | |
| NH white | 1.54 (1.33, 1.74) | 1.45 (1.38, 1.51) | 1.53 (1.38, 1.68) | 1.54 (1.29, 1.78) | -0.003 (-0.29, 0.29) | 0.861 |
| NH black | 1.44 (1.23, 1.65) | 1.36 (1.22, 1.50) | 1.50 (1.34, 1.65) | 1.14 (0.99, 1.29) | -0.30 (-0.54, -0.07) | 0.014 |
| Hispanic | 1.86 (1.60, 2.12) | 1.65 (1.43, 1.89) | 1.82 (1.64, 2.00) | 1.69 (1.45, 1.93) | -0.17 (-0.50, 0.15) | 0.368 |
| NH Asian | 1.59 (1.37, 1.81) | 1.52 (1.34, 1.69) | 1.75 (1.51, 2.00) | 1.41 (1.10, 1.71) | -0.18 (-0.53, 0.16) | 0.489 |
| Other race | 1.38 (0.92, 1.84) | 1.25 (0.96, 1.54) | 1.75 (1.32, 2.18) | 1.51 (1.13, 1.90) | 0.13 (-0.42, 0.68) | 0.408 |
| Processed meat (servings) | | | | | | |
| NH white | 0.99 (0.87, 1.10) | 1.06 (0.95, 1.16) | 1.07 (0.97, 1.18) | 1.04 (0.91, 1.16) | -0.05 (-0.18, 0.08) | 0.996 |
| NH black | 1.07 (0.97, 1.17) | 1.03 (0.88, 1.17) | 0.91 (0.82, 1.01) | 0.98 (0.88, 1.08) | -0.08 (-0.21, 0.05) | 0.024 |
| Hispanic | 0.77 (0.63, 0.91) | 0.72 (0.62, 0.83) | 0.70 (0.60, 0.80) | 0.72 (0.56, 0.88) | -0.05 (-0.25, 0.14) | 0.594 |
| NH Asian | 0.55 (0.43, 0.66) | 0.60 (0.42, 0.77) | 0.38 (0.26, 0.51) | 0.41 (0.26, 0.55) | -0.14 (-0.31, 0.03) | 0.046 |
| Other race | 0.87 (0.56, 1.18) | 0.87 (0.58, 1.17) | 1.15 (0.89, 1.42) | 0.81 (0.59, 1.03) | -0.06 (-0.41, 0.29) | 0.994 |
| Poultry (servings) | | | | | | |
| NH white | 1.26 (1.09, 1.44) | 1.52 (1.35, 1.70) | 1.38 (1.18, 1.59) | 1.35 (1.20, 1.50) | 0.09 (-0.12, 0.30) | 0.131 |
| NH black | 2.10 (1.90, 2.30) | 2.09 (1.88, 2.30) | 2.19 (1.96, 2.42) | 2.15 (1.93, 2.37) | 0.05 (-0.22, 0.33) | 0.066 |
| Hispanic | 1.68 (1.43, 1.92) | 1.85 (1.55, 2.14) | 1.77 (1.54, 2.00) | 1.60 (1.37, 1.83) | -0.08 (-0.39, 0.23) | 0.529 |
| NH Asian | 1.59 (1.37, 1.80) | 1.72 (1.45, 2.01) | 1.52 (1.29, 1.74) | 1.70 (1.26, 2.13) | 0.11 (-0.34, 0.56) | 0.807 |
| Other race | 1.34 (0.95, 1.73) | 1.64 (1.02, 2.26) | 1.99 (1.64, 2.34) | 1.58 (1.01, 2.15) | 0.23 (-0.40, 0.86) | 0.401 |
| Fish/seafood (servings) | | | | | | |

| | | | | | | |
|---|---|---|---|---|---|---|
| NH white | 0.52 (0.36, 0.69) | 0.61 (0.46, 0.76) | 0.52 (0.41, 0.64) | 0.54 (0.41, 0.67) | 0.02 (-0.17, 0.21) | 0.557 |
| NH black | 0.86 (0.57, 1.16) | 1.00 (0.69, 1.32) | 0.78 (0.62, 0.94) | 0.89 (0.72, 1.07) | 0.03 (-0.28, 0.35) | 0.514 |
| Hispanic | 0.66 (0.52, 0.80) | 0.64 (0.52, 0.76) | 0.65 (0.51, 0.79) | 0.69 (0.50, 0.87) | 0.03 (-0.18, 0.23) | 0.981 |
| NH Asian | 1.32 (1.12, 1.53) | 1.27 (0.99, 1.55) | 1.17 (0.84, 1.50) | 0.96 (0.70, 1.21) | -0.37 (-0.67, -0.07) | 0.230 |
| Other race | 0.65 (0.14, 1.16) | 0.67 (0.29, 1.06) | 0.44 (0.16, 0.72) | 0.46 (0.15, 0.76) | -0.19 (-0.74, 0.36) | 0.200 |
| Fish high in omega-3 fatty acids (servings) | | | | | | |
| NH white | 0.13 (0.07, 0.19) | 0.21 (0.15, 0.28) | 0.19 (0.12, 0.26) | 0.14 (0.09, 0.19) | 0.01 (-0.06, 0.08) | 0.374 |
| NH black | 0.19 (0.12, 0.26) | 0.16 (0.11, 0.21) | 0.18 (0.12, 0.24) | 0.18 (0.11, 0.26) | -0.01 (-0.10, 0.09) | 0.808 |
| Hispanic | 0.15 (0.10, 0.21) | 0.17 (0.11, 0.23) | 0.14 (0.10, 0.18) | 0.17 (0.09, 0.24) | 0.01 (-0.08, 0.10) | 0.946 |
| NH Asian | 0.42 (0.29, 0.55) | 0.55 (0.40, 0.69) | 0.49 (0.37, 0.62) | 0.37 (0.18, 0.56) | -0.06 (-0.27, 0.16) | 0.818 |
| Other race | 0.09 (0.02, 0.16) | 0.18 (0.05, 0.30) | 0.09 (-0.02, 0.20) | 0.07 (-0.01, 0.14) | -0.02 (-0.11, 0.07) | 0.930 |
| Fish low in omega-3 fatty acids (servings) | | | | | | |
| NH white | 0.37 (0.26, 0.48) | 0.40 (0.27, 0.54) | 0.34 (0.24, 0.44) | 0.41 (0.30, 0.51) | 0.03 (-0.10, 0.17) | 0.783 |
| NH black | 0.68 (0.41, 0.95) | 0.83 (0.51, 1.14) | 0.62 (0.46, 0.78) | 0.71 (0.56, 0.87) | 0.03 (-0.26, 0.32) | 0.424 |
| Hispanic | 0.50 (0.38, 0.61) | 0.47 (0.35, 0.59) | 0.53 (0.40, 0.67) | 0.53 (0.38, 0.69) | 0.04 (-0.14, 0.21) | 0.942 |
| NH Asian | 0.93 (0.77, 1.08) | 0.79 (0.60, 0.98) | 0.74 (0.49, 1.00) | 0.64 (0.37, 0.92) | -0.28 (-0.57, 0.004) | 0.151 |
| Other race | 0.55 (0.10, 0.99) | 0.51 (0.12, 0.90) | 0.35 (0.11, 0.59) | 0.40 (0.12, 0.68) | -0.15 (-0.63, 0.33) | 0.136 |
| Eggs (servings) | | | | | | |
| NH white | 0.54 (0.48, 0.59) | 0.58 (0.53, 0.63) | 0.61 (0.56, 0.67) | 0.70 (0.58, 0.81) | 0.16 (0.04, 0.28) | 0.017 |
| NH black | 0.62 (0.55, 0.69) | 0.65 (0.57, 0.74) | 0.62 (0.54, 0.71) | 0.73 (0.62, 0.84) | 0.11 (-0.02, 0.23) | 0.213 |
| Hispanic | 0.61 (0.52, 0.70) | 0.63 (0.54, 0.72) | 0.79 (0.70, 0.89) | 0.97 (0.76, 1.18) | 0.36 (0.16, 0.57) | 0.001 |

| | | | | | | |
|---|---|---|---|---|---|---|
| NH Asian | 0.59 (0.52, 0.67) | 0.71 (0.59, 0.83) | 0.75 (0.61, 0.90) | 0.89 (0.66, 1.11) | 0.29 (0.07, 0.51) | 0.002 |
| Other race | 0.65 (0.36, 0.94) | 0.67 (0.44, 0.89) | 0.43 (0.32, 0.53) | 0.54 (0.26, 0.82) | -0.11 (-0.48, 0.26) | 0.591 |
| Total dairy (servings) | | | | | | |
| NH white | 1.61 (1.54, 1.68) | 1.62 (1.54, 1.70) | 1.55 (1.48, 1.61) | 1.45 (1.38, 1.53) | -0.16 (-0.25, -0.06) | 0.001 |
| NH black | 1.07 (0.99, 1.16) | 1.09 (1.03, 1.15) | 0.98 (0.90, 1.06) | 1.02 (0.96, 1.09) | -0.05 (-0.15, 0.04) | 0.183 |
| Hispanic | 1.50 (1.41, 1.59) | 1.47 (1.36, 1.57) | 1.34 (1.26, 1.43) | 1.29 (1.20, 1.38) | -0.21 (-0.33, -0.09) | <0.001 |
| NH Asian | 1.09 (0.97, 1.21) | 1.13 (1.03, 1.22) | 1.02 (0.87, 1.18) | 1.14 (0.97, 1.31) | 0.05 (-0.13, 0.24) | 0.752 |
| Other race | 1.30 (1.12, 1.49) | 1.41 (1.15, 1.66) | 1.23 (1.06, 1.40) | 1.24 (1.06, 1.42) | -0.06 (-0.30, 0.17) | 0.303 |
| Milk products (servings) | | | | | | |
| NH white | 0.77 (0.71, 0.84) | 0.73 (0.68, 0.77) | 0.65 (0.60, 0.70) | 0.63 (0.56, 0.71) | -0.14 (-0.23, -0.05) | 0.001 |
| NH black | 0.48 (0.42, 0.54) | 0.44 (0.41, 0.48) | 0.41 (0.37, 0.44) | 0.41 (0.37, 0.46) | -0.07 (-0.13, 0.002) | 0.325 |
| Hispanic | 0.74 (0.66, 0.82) | 0.67 (0.56, 0.77) | 0.62 (0.54, 0.69) | 0.53 (0.46, 0.60) | -0.21 (-0.31, -0.11) | <0.001 |
| NH Asian | 0.63 (0.55, 0.71) | 0.63 (0.57, 0.69) | 0.62 (0.51, 0.73) | 0.72 (0.62, 0.83) | 0.10 (-0.03, 0.22) | 0.345 |
| Other race | 0.66 (0.55, 0.77) | 0.58 (0.43, 0.74) | 0.59 (0.44, 0.74) | 0.42 (0.24, 0.59) | -0.24 (-0.43, -0.06) | 0.079 |
| Cheese (servings) | | | | | | |
| NH white | 0.73 (0.67, 0.79) | 0.76 (0.71, 0.82) | 0.74 (0.70, 0.79) | 0.74 (0.68, 0.81) | 0.01 (-0.07, 0.09) | 0.770 |
| NH black | 0.55 (0.49, 0.60) | 0.58 (0.50, 0.66) | 0.52 (0.45, 0.59) | 0.56 (0.50, 0.62) | 0.01 (-0.07, 0.09) | 0.676 |
| Hispanic | 0.67 (0.60, 0.75) | 0.73 (0.64, 0.83) | 0.65 (0.59, 0.72) | 0.67 (0.59, 0.74) | -0.01 (-0.11, 0.09) | 0.097 |
| NH Asian | 0.35 (0.29, 0.40) | 0.43 (0.35, 0.51) | 0.30 (0.22, 0.37) | 0.34 (0.25, 0.43) | -0.003 (-0.10, 0.09) | 0.282 |
| Other race | 0.58 (0.44, 0.73) | 0.78 (0.53, 1.04) | 0.57 (0.49, 0.65) | 0.72 (0.58, 0.86) | 0.14 (-0.05, 0.32) | 0.657 |
| Nuts and seeds (servings) | | | | | | |

| | | | | | | |
|---|---|---|---|---|---|---|
| NH white | 0.81 (0.71, 0.92) | 0.81 (0.70, 0.93) | 0.84 (0.69, 0.98) | 0.86 (0.70, 1.02) | 0.04 (-0.13, 0.22) | 0.153 |
| NH black | 0.54 (0.42, 0.66) | 0.46 (0.37, 0.56) | 0.43 (0.33, 0.54) | 0.54 (0.39, 0.69) | 0.0004 (-0.18, 0.18) | 0.784 |
| Hispanic | 0.42 (0.31, 0.52) | 0.45 (0.31, 0.58) | 0.36 (0.29, 0.42) | 0.51 (0.36, 0.66) | 0.09 (-0.08, 0.26) | 0.826 |
| NH Asian | 0.81 (0.65, 0.98) | 0.85 (0.70, 1.00) | 0.92 (0.75, 1.09) | 0.86 (0.60, 1.12) | 0.05 (-0.24, 0.33) | 0.900 |
| Other race | 0.46 (0.26, 0.66) | 0.66 (0.31, 1.00) | 1.02 (0.48, 1.56) | 0.69 (0.42, 0.96) | 0.23 (-0.08, 0.54) | 0.164 |
| Added sugar (tsp) | | | | | | |
| NH white | 16.36 (15.30, 17.43) | 15.82 (15.00, 16.64) | 15.28 (14.48, 16.08) | 15.76 (14.68, 16.84) | -0.61 (-1.99, 0.76) | 0.185 |
| NH black | 18.48 (17.50, 19.47) | 18.23 (17.27, 19.19) | 17.02 (15.78, 18.27) | 17.29 (16.23, 18.35) | -1.16 (-2.46, 0.15) | 0.018 |
| Hispanic | 15.98 (14.56, 17.40) | 16.14 (15.09, 17.19) | 15.00 (14.23, 15.76) | 14.52 (13.23, 15.82) | -1.45 (-3.24, 0.34) | 0.035 |
| NH Asian | 11.41 (10.11, 12.72) | 10.52 (9.57, 11.47) | 9.71 (9.24, 10.17) | 10.50 (9.72, 11.28) | -0.91 (-2.31, 0.49) | 0.068 |
| Other race | 18.43 (15.35, 21.50) | 18.82 (15.45 22.20) | 15.47 (13.33, 17.61) | 17.77 (15.89, 19.65) | -0.61 (-3.91, 2.68) | 0.390 |
| **Nutrients (per day)** | | | | | | |
| Carbohydrate (g) | | | | | | |
| NH white | 243.22 (238.92, 247.53) | 234.47 (230.88, 238.07) | 228.48 (224.03, 232.92) | 227.91 (224.32, 231.50) | -15.32 (-20.45, -10.18) | <0.001 |
| NH black | 245.45 (241.17, 249.73) | 241.22 (236.51, 245.92) | 238.02 (232.18, 243.87) | 235.71 (231.57, 239.85) | -9.74 (-15.23, -4.25) | <0.001 |
| Hispanic | 251.24 (245.44, 257.03) | 247.37 (243.25, 251.48) | 245.75 (240.45, 251.04) | 243.46 (236.33, 250.59) | -7.78 (-16.19, 0.64) | 0.007 |
| NH Asian | 262.71 (257.23, 268.20) | 251.78 (245.92, 257.58) | 254.01 (250.10, 257.93) | 252.99 (248.15, 257.84) | -9.72 (-16.42, -3.02) | 0.019 |
| Other race | 259.16 (246.69, 271.64) | 248.83 (238.75, 258.91) | 223.14 (214.86, 231.41) | 232.91 (221.32, 244.49) | -26.25 (-41.87, -10.64) | <0.001 |
| Protein (g) | | | | | | |
| NH white | 76.71 (75.21, 78.20) | 80.03 (77.27, 82.79) | 79.65 (77.58, 81.72) | 77.28 (75.42, 79.13) | 0.57 (-1.61, 2.76) | 0.883 |
| NH black | 77.60 (76.07, 79.14) | 77.89 (75.93, 79.85) | 76.86 (74.96, 78.76) | 74.65 (72.99, 76.31) | -2.95 (-5.02, -0.88) | 0.001 |

| | | | | | | |
|---|---|---|---|---|---|---|
| Hispanic | 82.96 (81.51, 84.41) | 83.31 (80.55, 86.07) | 82.07 (80.36, 83.78) | 82.27 (79.32, 85.23) | -0.69 (-3.70, 2.33) | 0.439 |
| NH Asian | 84.43 (82.13, 86.74) | 86.67 (83.88, 89.46) | 86.85 (84.99, 88.71) | 83.33 (80.43, 86.23) | -1.11 (-4.50, 2.29) | 0.257 |
| Other race | 73.75 (70.74, 76.76) | 77.62 (72.98, 82.26) | 80.04 (74.73, 85.36) | 75.38 (72.31, 78.46) | 1.64 (-2.57, 5.85) | 0.532 |
| Saturated fat (g) | | | | | | |
| NH white | 24.15 (23.30, 25.00) | 25.11 (24.68, 25.54) | 26.71 (26.14, 27.29) | 27.35 (26.64, 28.07) | 3.20 (2.18, 4.23) | <0.001 |
| NH black | 22.84 (22.19, 23.50) | 23.57 (22.40, 24.74) | 24.35 (23.22, 25.47) | 24.59 (23.83, 25.35) | 1.75 (0.84, 2.66) | <0.001 |
| Hispanic | 22.85 (22.21, 23.49) | 23.28 (22.64, 23.92) | 24.06 (23.28, 24.85) | 23.99 (23.06, 24.93) | 1.14 (0.10, 2.18) | 0.010 |
| NH Asian | 19.14 (18.53, 19.74) | 20.47 (19.84, 21.10) | 20.86 (20.46, 21.27) | 22.05 (20.93, 23.16) | 2.91 (1.75, 4.07) | <0.001 |
| Other race | 22.22 (20.58, 23.86) | 24.15 (22.64, 25.67) | 26.22 (25.04, 27.41) | 25.75 (23.44, 28.07) | 3.54 (0.93, 6.14) | 0.011 |
| Monounsaturated fat (g) | | | | | | |
| NH white | 26.49 (25.89, 27.08) | 26.75 (26.36, 27.14) | 28.26 (27.68, 28.84) | 28.08 (27.47, 28.68) | 1.59 (0.81, 2.37) | <0.001 |
| NH black | 26.90 (26.24, 27.56) | 26.82 (26.01, 27.63) | 27.90 (27.03, 28.77) | 27.68 (26.92, 28.43) | 0.78 (-0.14, 1.70) | 0.005 |
| Hispanic | 25.76 (24.72, 26.79) | 25.22 (24.66, 25.79) | 26.25 (25.49, 27.01) | 25.60 (24.65, 26.54) | -0.16 (-1.45, 1.13) | 0.483 |
| NH Asian | 23.74 (22.94, 24.53) | 25.10 (24.05, 26.15) | 25.31 (24.68, 25.93) | 25.25 (24.41, 26.08) | 1.51 (0.45, 2.57) | 0.012 |
| Other race | 25.60 (23.01, 28.19) | 25.39 (24.16, 26.62) | 29.67 (27.97, 31.36) | 27.71 (25.81, 29.61) | 2.11 (-0.84, 5.06) | 0.018 |
| Polyunsaturated fat (g) | | | | | | |
| NH white | 17.79 (17.47, 18.12) | 18.18 (17.79, 18.57) | 18.39 (17.80, 18.98) | 19.32 (18.46, 20.17) | 1.52 (0.68, 2.36) | <0.001 |
| NH black | 18.73 (18.25, 19.21) | 18.19 (17.55, 18.83) | 18.87 (18.30, 19.45) | 20.18 (19.39, 20.96) | 1.45 (0.60, 2.29) | <0.001 |
| Hispanic | 17.20 (16.51, 17.89) | 17.20 (16.53, 17.87) | 17.14 (16.54, 17.75) | 17.61 (16.74, 18.49) | 0.41 (-0.61, 1.43) | 0.270 |
| NH Asian | 16.94 (16.16, 17.71) | 17.87 (16.96, 18.78) | 17.33 (16.39, 18.27) | 17.81 (16.96, 18.65) | 0.87 (-0.18, 1.92) | 0.233 |
| Other race | 17.46 (15.26, 19.65) | 17.16 (15.59, 18.72) | 18.78 (17.47, 20.09) | 18.48 (17.54, 19.41) | 1.02 (-1.17, 3.22) | 0.156 |

| | | | | | | |
|---|---|---|---|---|---|---|
| Omega-3 fatty acid (g) | | | | | | |
| NH white | 0.08<br>(0.07, 0.09) | 0.09<br>(0.08, 0.10) | 0.09<br>(0.08, 0.10) | 0.09<br>(0.08, 0.10) | 0.008<br>(-0.01, 0.02) | 0.737 |
| NH black | 0.13<br>(0.11, 0.15) | 0.12<br>(0.10, 0.14) | 0.11<br>(0.10, 0.12) | 0.11<br>(0.09, 0.12) | -0.02<br>(-0.04, 0.003) | 0.929 |
| Hispanic | 0.10<br>(0.09, 0.11) | 0.09<br>(0.08, 0.11) | 0.10<br>(0.08, 0.11) | 0.09<br>(0.08, 0.11) | -0.01<br>(-0.02, 0.01) | 0.626 |
| NH Asian | 0.13<br>(0.11, 0.15) | 0.13<br>(0.10, 0.15) | 0.12<br>(0.10, 0.15) | 0.10<br>(0.07, 0.12) | -0.04<br>(-0.06, 0.01) | 0.533 |
| Other race | 0.09<br>(0.07, 0.12) | 0.10<br>(0.07, 0.13) | 0.09<br>(0.07, 0.11) | 0.08<br>(0.06, 0.09) | -0.01<br>(-0.04, 0.01) | 0.358 |
| Cholesterol (mg) | | | | | | |
| NH white | 255.89<br>(247.12, 264.66) | 275.77<br>(266.79, 284.75) | 286.33<br>(275.26, 297.40) | 292.17<br>(276.23, 308.12) | 36.28<br>(19.61, 52.95) | <0.001 |
| NH black | 292.00<br>(281.88, 302.13) | 299.25<br>(290.04, 308.46) | 304.34<br>(285.84, 322.83) | 299.57<br>(288.01, 311.13) | 7.57<br>(-6.51, 21.65) | 0.266 |
| Hispanic | 293.62<br>(279.48, 307.75) | 293.73<br>(282.84, 304.63) | 317.60<br>(302.28, 332.93) | 319.49<br>(295.94, 343.03) | 25.87<br>(0.72, 51.03) | 0.008 |
| NH Asian | 249.23<br>(237.86, 260.60) | 264.18<br>(248.75, 279.61) | 277.34<br>(258.31, 296.36) | 274.11<br>(249.17, 299.04) | 24.88<br>(-0.22, 49.98) | 0.049 |
| Other race | 255.54<br>(222.13, 288.95) | 276.39<br>(236.75, 316.02) | 288.26<br>(258.71, 317.80) | 274.10<br>(245.98, 302.22) | 18.56<br>(-21.50, 58.62) | 0.728 |
| Sodium (g) | | | | | | |
| NH white | 3342.58<br>(3305.43, 3379.72) | 3391.36<br>(3334.89, 3447.82) | 3403.91<br>(3327.86, 3479.96) | 3370.29<br>(3276.99, 3463.59) | 27.71<br>(-64.28, 119.70) | 0.572 |
| NH black | 3327.63<br>(3255.45, 3399.81) | 3317.00<br>(3261.89, 3372.11) | 3361.73<br>(3272.62, 3450.84) | 3299.88<br>(3184.98, 3414.77) | -27.75<br>(-152.04, 96.54) | 0.517 |
| Hispanic | 3372.67<br>(3309.10, 3436.25) | 3315.54<br>(3224.31, 3406.77) | 3351.3<br>(3265.06, 3437.55) | 3372.45<br>(3143.62, 3601.28) | -0.22<br>(-217.78, 217.34) | 0.402 |
| NH Asian | 3895.77<br>(3800.79, 3990.75) | 3884.84<br>(3752.54, 4017.14) | 4024.29<br>(3755.83, 4292.75) | 3639.34<br>(3536.51, 3742.18) | -256.43<br>(-384.65, -128.21) | 0.009 |
| Other race | 3357.84<br>(3183.54, 3532.13) | 3267.14<br>(3048.96, 3485.31) | 3551.27<br>(3339.25, 3763.29) | 3247.14<br>(3080.60, 3413.68) | -110.70<br>(-331.78, 110.38) | 0.876 |
| Fiber (g) | | | | | | |
| NH white | 17.46<br>(16.66, 18.26) | 16.56<br>(15.84, 17.27) | 16.80<br>(16.03, 17.56) | 15.78<br>(15.19, 16.37) | -1.68<br>(-2.59, -0.77) | <0.001 |
| NH black | 14.67<br>(13.88, 15.45) | 14.39<br>(13.83, 14.94) | 14.48<br>(13.71, 15.25) | 14.29<br>(13.62, 14.96) | -0.37<br>(-1.32, 0.57) | 0.621 |

| | | | | | | |
|---|---|---|---|---|---|---|
| Hispanic | 18.20<br>(17.17, 19.23) | 18.50<br>(17.16, 19.84) | 18.48<br>(17.65, 19.32) | 18.46<br>(17.17, 19.75) | 0.26<br>(-1.25, 1.77) | 0.644 |
| NH Asian | 20.07<br>(19.07, 21.07) | 20.46<br>(19.25, 21.68) | 20.78<br>(19.62, 21.93) | 21.07<br>(19.34, 22.80) | 1.00<br>(-0.84, 2.83) | 0.238 |
| Other race | 16.49<br>(15.08, 17.90) | 15.33<br>(13.05, 17.62) | 15.77<br>(14.17, 17.37) | 15.14<br>(13.89, 16.38) | -1.35<br>(-3.08, 0.38) | 0.606 |

[a] *p* value from trend tests by modeling survey period as a continuous variable.

**Reference:**


1. Reedy J; Lerman JL; Krebs-Smith SM; Kirkpatrick SI; Pannucci TE; Wilson MM; Subar AF; Kahle LL; Tooze JA. Evaluation of the Healthy Eating Index-2015. *J Acad Nutr Diet* **2018**, *118*, 1622-1633.
2. National Cancer Institute. Division of Cancer Control and Population Sciences. Population Ratio Method. . Available online: https://epi.grants.cancer.gov/hei/population-ratio-method.html (accessed on June 15).
3. Tooze JA; Midthune D; Dodd KW; Freedman LS; Krebs-Smith SM; Subar AF; Guenther PM; Carroll RJ; Kipnis VA. A new statistical method for estimating the usual intake of episodically consumed foods with application to their distribution. *J Am Diet Assoc* **2006**, *106*, 1575–1587.
4. Herrick KA; Rossen LM; Parsons R; Dodd KW. Estimating usual dietary intake from National Health and Nutrition Examination Survey data using the National Cancer Institute method. 2018. *Vital Health Stat 2.* **2018**, *178*, 1-6.
5. Bailey, R.L.; Dodd, K.W.; Goldman, J.A.; Gahche, J.J.; Dwyer, J.T.; Moshfegh, A.J.; Sempos, C.T.; Picciano, M.F. Estimation of total usual calcium and vitamin D intakes in the United States. *The Journal of nutrition* **2010**, *140*, 817-822.
6. National Cancer Institute. Usual Dietary Intakes: Details of the Method. Available online: https://epi.grants.cancer.gov/diet/usualintakes/details.html (accessed on June 25).
